# Correlation between leaf epicuticular wax composition and structure, physio‐biochemical traits and drought resistance in glaucous and non‐glaucous near‐isogenic lines of rye

**DOI:** 10.1111/tpj.15428

**Published:** 2021-08-20

**Authors:** Kamila Laskoś, Ilona M. Czyczyło‐Mysza, Michał Dziurka, Angelika Noga, Magdalena Góralska, Jakub Bartyzel, Beata Myśków

**Affiliations:** ^1^ The Franciszek Górski Institute of Plant Physiology Polish Academy of Sciences Niezapominajek 21 30‐239 Kraków Poland; ^2^ Department of Plant Genetics, Breeding and Biotechnology West‐Pomeranian University of Technology Słowackiego 17 71‐434 Szczecin Poland; ^3^ Department of Applied Nuclear Physics Faculty of Physics and Applied Computer Science AGH University of Science and Technology Mickiewicza 3 30‐059 Kraków Poland

**Keywords:** abiotic stress, glaucousness, epicuticular wax, photosynthetic pigments, chlorophyll *a* fluorescence, tocochromanols, malondialdehyde, yield, winter rye

## Abstract

The objective of this research was to investigate the differences between glaucous and non‐glaucous near‐isogenic lines (NILs) of winter rye (*Secale cereale* L.) in terms of epicuticular wax layer properties (weight, composition, and crystal morphology), selected physiological and biochemical responses, yield components, above‐ground biomass, and plant height under soil drought stress. An important aspect of this analysis was to examine the correlation between the above characteristics. Two different NIL pairs were tested, each consisting of a typical glaucous line and a non‐glaucous line with a recessive mutation. The drought experiment was conducted twice (2015–2016). Our study showed that wax accumulation during drought was not correlated with higher leaf hydration and glaucousness. Environmental factors had a large impact on the response of the lines to drought in individual years, both in terms of physiological and biochemical reactions, and the composition of epicuticular leaf wax. The analysed pairs displayed significantly different responses to drought. Demonstration of the correlation between the components of rye leaf wax and the physiological and biochemical parameters of rye NILs is a significant achievement of this work. Interestingly, the study showed a correlation between the wax components and the content of photosynthetic pigments and tocopherols, whose biosynthesis, similarly to the biosynthesis of wax precursors, is mainly located in chloroplasts. This suggests a relationship between wax biosynthesis and plant response to various environmental conditions and drought stress.

## Significance Statement

No previous studies have focused on the role of wax cover in the resistance of rye plants to abiotic factors, particularly drought resistance; thus, our study on wax bloom is a recent research path for this cereal. Our results demonstrated a correlation between the wax components and the content of photosynthetic pigments and tocopherols, the biosynthesis of which are similar to the biosynthesis of wax precursors, which occurs mainly in chloroplasts.

## INTRODUCTION

Drought resistance is a particularly desirable feature of crops, such as cereals. Drought stress is perceived as the main factor limiting the improvement of crop productivity worldwide (Araus et al., 2008; Boyer, 1982). Water deficit in the coming years is expected to increase with global warming (Wheeler and von Braun, 2013). It has become important to search for features that determine whether a species is resistant to drought or sensitive, which is why some research groups have become interested in the wax surface of the above‐ground plant organs, i.e. the cuticle. There are well‐described cuticle functions, such as protection against radiation, including ultraviolet, also affecting plant–insect interactions, protection against non‐stomatal water loss or pathogens (Jenks et al., 1994; Holmes and Keiller, 2002; Eigenbrode and Jetter, 2002; Goodwin and Jenks, 2005; Gorb, 2005; Serrano et al., 2014; Wang et al., 2015a). Many studies have reported the relationship between plant wax characteristics and drought resistance, e.g. in maize (Meeks et al., 2012), wheat (Bi et al., 2017; Clarke and Richards, 1988; Guo et al., 2016), rice (Islam et al., 2009), cotton (Bondada and Oosterhuis, 2002), alfalfa (Ni et al., 2012), peas (Sánchez et al., 2001), and Arabidopsis (Yang et al., 2011). Only a few studies concerning the rye wax cover analysed its chemical composition (Ji and Jetter, 2008; Streibl et al., 1974; Sun et al., 2020; Tulloch and Hoffman, 1974). However, no work has focused on its role in the resistance of rye plants to abiotic factors, particularly drought resistance, which makes our study on wax bloom a different aspect of research for this cereal.

The cuticle is a plant lipid layer consisting of cutin and intra‐ and epicuticular waxes. Epicuticular wax forms the outermost hydrophobic layer of the cuticle and consists of a complex mixture of very long chain fatty acids (VLCFAs) and their derivatives, including hydrocarbons, wax esters, alcohols, aldehydes, ketones, terpenes, and flavones. The composition of this layer varies between plants, organs, and cells, and depends on the development stage or environmental factors (Baker and Hunt, 1981; Jenks et al., 2000; Kolattukudy, 1970; von Wettstein‐Knowles, 2016). The initiation of wax formation occurs in epidermal plastids, where *de novo* biosynthesis of C_16_ and C_18_ fatty acids (FAs) takes place. The produced FAs are transported from the plastid stroma to the endoplasmic reticulum membrane and are elongated to VLCFAs C_20_–C_34_ by FA elongation multienzyme complexes. Then, VLCFAs can be transformed via three biosynthetic pathways: (i) acyl reduction pathway generating primary alcohols and wax esters; (ii) decarbonylation pathway, leading to the formation of aldehydes, alkanes, secondary alcohols, and ketones; and (iii) β‐diketones biosynthesis pathway, used by *Triticum* species (Tulloch, 1973; von Wettstein‐Knowles, 2016). Epicuticular waxes on most plant surfaces accumulate as a smooth and transparent layer. However, many plant waxes crystallize on plant surfaces, forming a structure visible as a bluish cover. Plants that exhibit this feature are called glaucous or waxy and the wax layer is referred to as a wax bloom (glaucousness). The contrasting form is termed glossy, non‐glaucous, eceriferum, bloomless, or waxless (Hen‐Avivi et al., 2016). These surfaces observed under a scanning electron microscope (SEM) show the presence of crystalloid structures specific to plants and their organs (Jenks and Ashworth, 1999). Leaf glaucousness is a morphological characteristic of plants that differentiates them in terms of features, such as reduced epidermic conductivity and surface light reflectance (Biswal and Kohli, 2013). The level of glaucousness is not directly associated with the amount of wax itself (Araus et al., 1991; Febrero et al., 1998; Larsson and Svenningsson, 1986), but rather with the deposition and orientation of wax crystals on the cuticle of photosynthetic surfaces (Yoshiya et al., 2011). A different structure of wax crystals in glaucous and non‐glaucous plants indicates their different chemical composition (Bi et al., 2017; Wang et al., 2015b). Willick et al. (2018) described the microstructure and chemical composition of the wax coating of wheat flag leaves and indicated that this feature could be a prospective potential marker for drought resistance. Glaucous plants, easily distinguishable from others, can be a valuable source of genetic information for breeders. An increase in the reflectance of the flag leaf of glaucous wheat lines was observed in water deficiency conditions (Johnson et al., 1983); a glaucous wheat line also showed more desirable yield‐related traits in terms of drought resistance compared with a non‐glaucous line (Richards et al., 1986). Only few studies demonstrated differences in the physiological aspects of plants with different wax bloom patterns under water deficit (Guo et al., 2016; Su et al., 2020). Glaucous and non‐glaucous plants were compared mainly in terms of wax composition (Bi et al., 2017) or yield and water relations (Clarke and Richards, 1988; Febrero et al., 1998; Guo et al., 2016; Johnson et al., 1983; Merah et al., 2000; Willick et al., 2018). However, it has rarely been explored whether these plants may also differ in other features that could be affected by different wax bloom under drought, e.g. the content of photosynthetic pigments, tocopherols, phenolic compounds, or soluble sugars. An increasing number of studies have indicated the important role of genes and transcription factors of wax biosynthesis in higher plant drought resistance (Xue et al., 2017). Jiang et al. (2009), studied WXP1 (ethylene‐responsive element‐binding transcription factor) in transgenic alfalfa and pointed out that *WXP1* overexpression not only led to enhanced drought resistance and wax accumulation, but might also be involved in other physiological responses important for improved drought resistance. Further research on plant reactions to drought stress is necessary to understand better the sources of drought resistance in rye and other plants, and potentially discover some pleiotropic effects in further studies. The objective of this research was to investigate the differences between glaucous and non‐glaucous near‐isogenic lines (NILs) of rye in terms of epicuticular wax layer properties (weight, composition, and crystal morphology) and selected physiological and biochemical responses, yield components, above‐ground biomass, and plant height under soil drought stress. An important aspect of this analysis was to examine the correlation between the above characteristics.

## RESULTS

### Determining weather conditions during drought experiments

**Figure 1:**
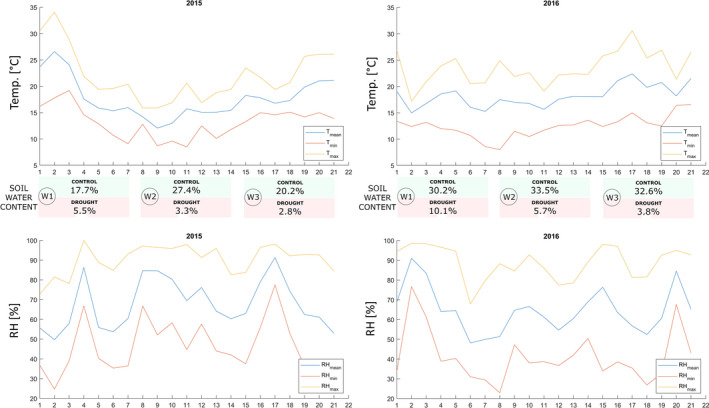
Weather conditions during a 3‐week drought period During the first (W1), second (W2), and third (W3) week of drought in the first (2015) and second year (2016) of experiment: daily average (*T*_mean_), minimum (*T*_min_), and maximum (*T*_max_) temperature; daily average (RH_mean_), minimum (RH_min_), and maximum (RH_max_); relative humidity; and mean soil water content [%].

Weather status was compared in the 2‐year experiment during drought periods in 2015 and 2016, including maximum, mean, and minimum temperatures, as well as relative humidity in Cracow (Figure 1, Table S1). It can be concluded from the comparison of the 2015 and 2016 drought periods that there were no statistically significant differences between the mean values (17.7 and 18.2°C, respectively) in the study periods or minimum temperatures (8.5 and 8.0°C, respectively). Only the maximum temperature in 2015 (34.1°C) was significantly higher than in 2016 (30.6°C) (Figure 1). With daily fluctuations in the relative humidity values, no significant differences in the measured parameters were observed (Figure 1). In both years of the experiment, the difference in soil moisture under both treatments remained very pronounced and persisted in each week of stress; however, moisture content measured for both treatments in 2015 was lower than in 2016 (Figure 1). It is worth noting that soil moisture was lower both in control and drought conditions, particularly at the beginning of the drought period, when higher maximum temperature were recorded (Figure 1).

### Differentiation of biochemical, physiological, and morphological characteristics for glaucous and non‐glaucous rye NILs subjected to drought stress

The analysis of variance showed significant differences resulting from the interaction of line × wax cover type (occurrence or absence of mutations) × treatment among most of the analysed parameters in the 2‐year experiment (Table 1). The lack of significance of interactions between these effects in both years was demonstrated in the context of α‐ and γ‐tocotrienol content and plant height (Table 1). Interaction of the effects of line × wax cover × treatment at high (*P* ≤ 0.01) and highest (*P* ≤ 0.001) level of significance in both years of the experiment exerted an effect on the content of individual carotenoids [violaxanthin (Viol), lutein (Lut), zeaxanthin (Zea), β‐carotene (β‐car)] and their sum, chlorophyll *a* (ChlA) and *b* (ChlB), and total chlorophyll (TChl), γ‐ and δ‐tocopherol (γ‐T and δ‐T) and ChlA fluorescence parameters [amount of excitation energy trapped in PSII reaction centres (TRo/CSm), light energy absorption (ABS/CSm)] (Table 1).

**Table 1:** Interaction between genotype (line), wax layer type (glaucous versus non‐glaucous) and treatment (control versus drought) over the 2 years of the experiment

Trait	Year	*F*‐value	*P*‐value	Trait	Year	*F*‐value	*P*‐value
TPC	2015	1.20	0.2822	γ‐T	2015	19.19**	0.0005
	2016	70.56**	0.0000		2016	17.58**	0.0008
SSC	2015	1.78	0.1918	δ‐T	2015	67.80**	0.0000
	2016	181.01**	0.0000		2016	12.32*	0.0032
Viol	2015	130.08**	0.0000	MDA	2015	14/93*	0/0014
	2016	30.33**	0.0001		2016	1.21	0/2881
Lut	2015	1713.52**	0.0000	PI	2015	4.04	0.0533
	2016	96.12**	0.0000		2016	7.13*	0.0095
Zea	2015	1079.61**	0.0000	ABS/CSm	2015	12.21*	0.0015
	2016	19.26**	0.0005		2016	10.73*	0.0017
β‐car	2015	844.58**	0.0000	TRo/CSm	2015	13.98**	0.0008
	2016	136.54**	0.0000		2016	8.27*	0.0054
ChlA	2015	13.12**	0.0010	ETo/CSm	2015	11.02*	0.0023
	2016	76.29**	0.0000		2016	6.88***	0.0108
ChlB	2015	7.12***	0.0120	RC/CSm	2015	11.85*	0.0017
	2016	5.79***	0.0222		2016	6.56***	0.0127
Ratio ChlA/B	2015	363.68**	0.0000	DIo/CSm	2015	5.79***	0.0222
	2016	1.38	0.2482		2016	34.88**	0.0000
TChl	2015	11.72*	0.0018	GN	2015	0.19	0.6671
	2016	18.60**	0.0002		2016	4.73***	0.0321
Car	2015	18.80**	0.0001	GW	2015	0.73	0.3965
	2016	26.25**	0.0000		2016	15.02**	0.0002
α‐T3	2015	0.03	0.8657	TGW	2015	38.90**	0.0000
	2016	1.84	0.1955		2016	4.54***	0.0357
δ‐T3	2015	4.01	0.0625	B	2015	0.10	0.7586
	2016	24.24**	0.0002		2016	9.08*	0.0033
γ‐T3	2015	1.82	0.1956	PH	2015	1.23	0.2734
	2016	3.43	0.0837		2016	3.82	0.0536
α‐T	2015	5.28***	0.0354	δ^13^C	2015	15.36*	0.0012
	2016	0.12	0.7308		2016	7.23***	0.0162
β‐T	2015	7.86***	0.0127				
	2016	2.33	0.1477				

ABS/CSm, light energy absorption; B, above‐ground biomass; Car, total carotenoid content; ChlA, chlorophyll *a* content; ChlB, chlorophyll *b* content; DIo/CSm, amount of energy dissipated from PSII; ETo/CSm, amount of energy used for electron transport; GN, grain number per plant; GW, grain weight per plant; Lut, lutein content; MDA, malondialdehyde content; P.I., overall performance index of photosystem II (PSII) photochemistry; PH, plant height with awns; ratio ChlA/ChlB, ratio of chlorophyll *a* and *b* content; RC/CSm, number of active reaction centres; SSC, soluble sugar content; TChl, total chlorophyll content; TGW, thousand grain weight; TPC, total phenolic content; TRo/CSm, amount of excitation energy trapped in PSII reaction centres; Viol, violaxanthin content; Zea, zeaxanthin content; α‐T, α‐tocopherol; α‐T3, α‐tocotrienol; β‐car, β‐carotene content; β‐T, β‐tocopherol; γ‐T, γ‐tocopherol; γ‐T3, γ‐tocotrienol; δ^13^C, carbon isotope discrimination; δ‐T, δ‐tocopherol; δ‐T3 content of δ‐tocotrienol.

Significance levels:

* *P* ≤ 0.05;

** *P* ≤ 0.01;

*** *P* ≤ 0.001.

### Pearson’s correlation analysis

Pearson’s correlation analysis, conducted separately for each year, revealed significant negative and positive correlations, but also no correlation between leaf wax components of rye NILs and biochemical and physiological features, yield, plant height, and above‐ground biomass (Table 2, Table S2). The analysis for the first year showed a significant positive correlation (*P* ≤ 0.05) between wax load and α‐tocotrienol content in leaves, plant biomass, and odd‐chain hydrocarbon contents and their total content and, lastly, between esters and the value of δ^13^C carbon isotope discrimination (Table 2). Significant negative correlations (*P* ≤ 0.05) were found between FA and soluble sugar contents (SSC) and photosynthetic pigments [ChlA, TChl, and Car (carotenoids sum)] in rye leaves (Table 2). The analysis of the results from 2016 showed that the content of esters correlated significantly positively with tocopherols (β‐T, γ‐T [*P* ≤ 0.01], and δ‐T [*P* ≤ 0.05]), and negatively with leaf hydration, SSCs (*P* ≤ 0.01), ChlA content, ChlA/B ratio, chlorophyll a fluorescence (Car and FC) parameters [ABS/CSm, Tro/CSm, amount of energy used for electron transport (Eto/CSm), number of active reaction centres (RC/CSm)] (*P* ≤ 0.05) (Table 2). Wax load on flag leaf correlated negatively with ChlB content (*P* ≤ 0.05) (Table 2). No correlation was found between the content of wax component and δ‐ and γ‐tocotrienols, α‐T, malondialdehyde (MDA), carotenoids (Viol, Lut, Zea, β‐car) as well as total phenolic content (TPC), yield [grain number per plant (GN), grain weight per plant (GW), thousand grain weight (TGW)], plant height, and two FC parameters [overall performance index of PSII photochemistry (PI), amount of energy dissipated from PSII (DIo/CSm)] (Table 2).

**Table 2:** Selected correlation coefficients between leaf epicuticular wax (wax load and fractions content) and biochemical and physiological parameters and above‐ground biomass of rye near‐isogenic lines obtained in separate analyses for 2015 and 2016

Variables	Leaf epicuticular wax							
	Year	Wax load	Hydrocarbons			Primary alcohols	Fatty acids	Esters
			Sum	Even‐chain	Odd‐chain			
H	2015	−0.41	0.39	0.27	0.4	ND	–0.66	–0.4
	2016	−0.52	−0.03	−0.46	0.2	0.35	0.28	−0.82***
δ^13^C	2015	0.6	−0.26	−0.03	−0.3	ND	0.68	0.72***
	2016	0.26	0.36	0.1	0.44	0.24	−0.66	−0.06
α‐T3	2015	0.74***	−0.39	−0.2	−0.42	ND	0.22	0.53
	2016	−0.15	0.04	0.34	−0.12	−0.04	−0.59	0.31
δ‐T	2015	0.48	−0.45	−0.32	−0.47	ND	0.65	0.51
	2016	0.53	−0.06	0.36	−0.28	−0.4	−0.17	0.74***
β‐T	2015	0.4	−0.28	−0.14	−0.31	ND	−0.29	0.32
	2016	0.56	−0.18	0.32	−0.41	−0.54	−0.05	0.9^a^
γ‐T	2015	0.7	−0.55	−0.33	−0.58	ND	0.49	0.59
	2016	0.48	−0.06	0.33	−0.25	−0.57	0.09	0.85^a^
SSC	2015	−0.43	0.13	−0.06	0.16	ND	−0.8***	−0.57
	2016	−0.49	−0.13	−0.54	0.11	0.48	0.26	−0.86^a^
ChlA	2015	−0.34	0.31	0.17	0.33	ND	−0.73***	−0.52
	2016	−0.55	−0.02	−0.37	0.17	0.58	0.04	−0.74***
ChlB	2015	−0.37	0.34	0.19	0.37	ND	−0.67	−0.54
	2016	−0.82***	0.53	0.6	0.41	0.31	−0.26	0.03
Ratio ChlA/B	2015	0.01	0.11	0.16	0.1	ND	−0.45	0.02
	2016	−0.3	−0.24	−0.64	0.01	0.33	0.23	−0.81***
TChl	2015	−0.35	0.32	0.17	0.34	ND	−0.72***	−0.53
	2016	−0.68	0.1	−0.2	0.24	0.58	−0.02	−0.65
Car	2015	−0.29	0.31	0.19	0.32	ND	−0.73***	−0.47
	2016	−0.41	−0.18	−0.53	0.03	0.55	0.09	−0.76***
B	2015	−0.58	0.77***	0.7	0.78***	ND	0.04	−0.18
	2016	−0.28	−0.37	−0.49	−0.25	0.49	0.02	−0.42
ABS/CSm	2015	−0.19	0.43	0.45	0.42	ND	−0.35	−0.04
	2016	−0.63	0.1	−0.29	0.3	0.38	0.22	−0.72***
TRo/CSm	2015	−0.18	0.41	0.44	0.4	ND	−0.37	−0.04
	2016	−0.61	0.09	−0.32	0.29	0.4	0.19	−0.75***
ETo/CSm	2015	−0.22	0.44	0.44	0.43	ND	−0.4	−0.08
	2016	−0.6	0.08	−0.34	0.29	0.47	0.17	−0.77***
RC/CSm	2015	−0.27	0.37	0.35	0.37	ND	−0.48	−0.17
	2016	−0.63	0.07	−0.33	0.27	0.45	0.14	−0.77***

ABS/CSm, light energy absorption; B, above‐ground biomass; Car, total carotenoids content; ChlA, chlorophyll *a* content; ChlB, chlorophyll *b* content; ETo/CSm, amount of energy used for electron transport; H, flag leaf hydration; ND, no data; ratio ChlA/ChlB, ratio of chlorophyll *a* and *b* content; RC/CSm, number of active reaction centres; SSC, soluble sugar content; TChl, total chlorophyll content; TRo/CSm, amount of excitation energy trapped in PSII reaction centres; α‐T3, α‐tocotrienol; β‐T, β‐tocopherol; γ‐T, γ‐tocopherol; δ^13^C, carbon isotope discrimination; δ‐T, δ‐tocopherol.

Significance levels:

* *P* ≤ 0.05;

** *P* ≤ 0.01;

*** *P* ≤ 0.001.

### Differentiation of epicuticular wax structure based on SEM analysis

**Figure 2:**
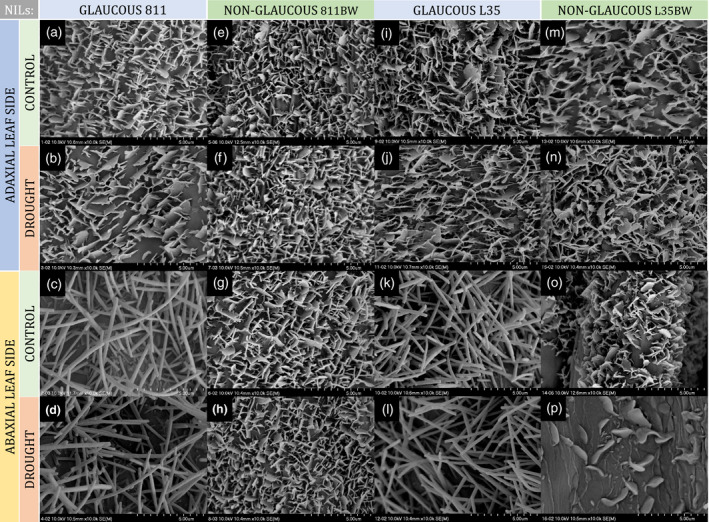
Scanning electron microscopy images of wax crystals on the surfaces of the adaxial and abaxial leaf side of rye near‐isogenic lines (NILs). Glaucous lines (a–d) 811 and (i–l) L35, and non‐glaucous lines (e–h) 811bw and (m–p) L35bw; under control and drought conditions. Image scale: 5 μm.

SEM analysis of the adaxial (upper, topside) and abaxial (lower, underside) leaf surface of rye NILs showed differences in wax crystal shapes between the non‐glaucous and glaucous lines in both tested pairs. The abaxial surface of the leaves was the differentiating side for both lines and treatments (Figure 2). Wax crystalloids occurred on both flag leaf sides of all rye NILs, and two primary crystalloid types were identified: platelets (Figure 2a,b,e–j,m–p) and tubules (Figure 2c,d,k,l), according to the classification of Barthlott et al. (1998); they were not oriented in any easily noticeable pattern. Under control conditions, both the upper and lower leaf surfaces of the non‐glaucous lines 811bw and L35bw were covered with platelets (Figure 2e,g,m,o). Platelet appearance, flat and irregularly shaped with a sinuate margin, meant classification into the group of irregular wax crystalloid platelets. Under drought, the same structures on both leaf surfaces were observed for the line 811bw (Figure 2f,h). Platelet‐like crystalloids observed during drought on the abaxial leaf area of the non‐glaucous L35bw line differed from platelets observed on the adaxial surface (Figure 2n,p). However, thicker structures (Figure 2p) on the surface with an entire margin were significantly less abundant than adaxial crystalloids (Figure 2n); it is difficult to classify them into a specific platelet subgroup. Under both treatments, the adaxial flag leaf area of the glaucous NILs 811 and L35 was also covered with platelets of the same shape as in the non‐glaucous lines (Figure 2a,b,i,j). However, the epicuticular wax formed tubule‐shaped crystals on the abaxial surface, which were long, terminally hollowed tubules with few acute‐angle branches (Figure ,k,l). The outer diameter of tubules of the glaucous lines 811 and L35 under control conditions was 0.14–0.39 and 0.14–0.37 µm, respectively (Table 2). These measurements did not change significantly during drought and amounted to 0.14–0.40 µm in line 811 and a slightly narrowed range of measurements of 0.17–0.34 µm in line L35 (Table 3).

**Table 3:** Outer diameter of glaucous line tubules under control and drought conditions in rye near‐isogenic lines

Line	Treatment	Mean ±SD (µm)	Min (µm)	Max (µm)
811	Control	0.26±0.05	0.14	0.39
811	Drought	0.25±0.05	0.14	0.40
L35	Control	0.25±0.04	0.14	0.37
L35	Drought	0.24±0.03	0.17	0.34

### Leaf hydration and the amount and components of wax

**Figure 3:**
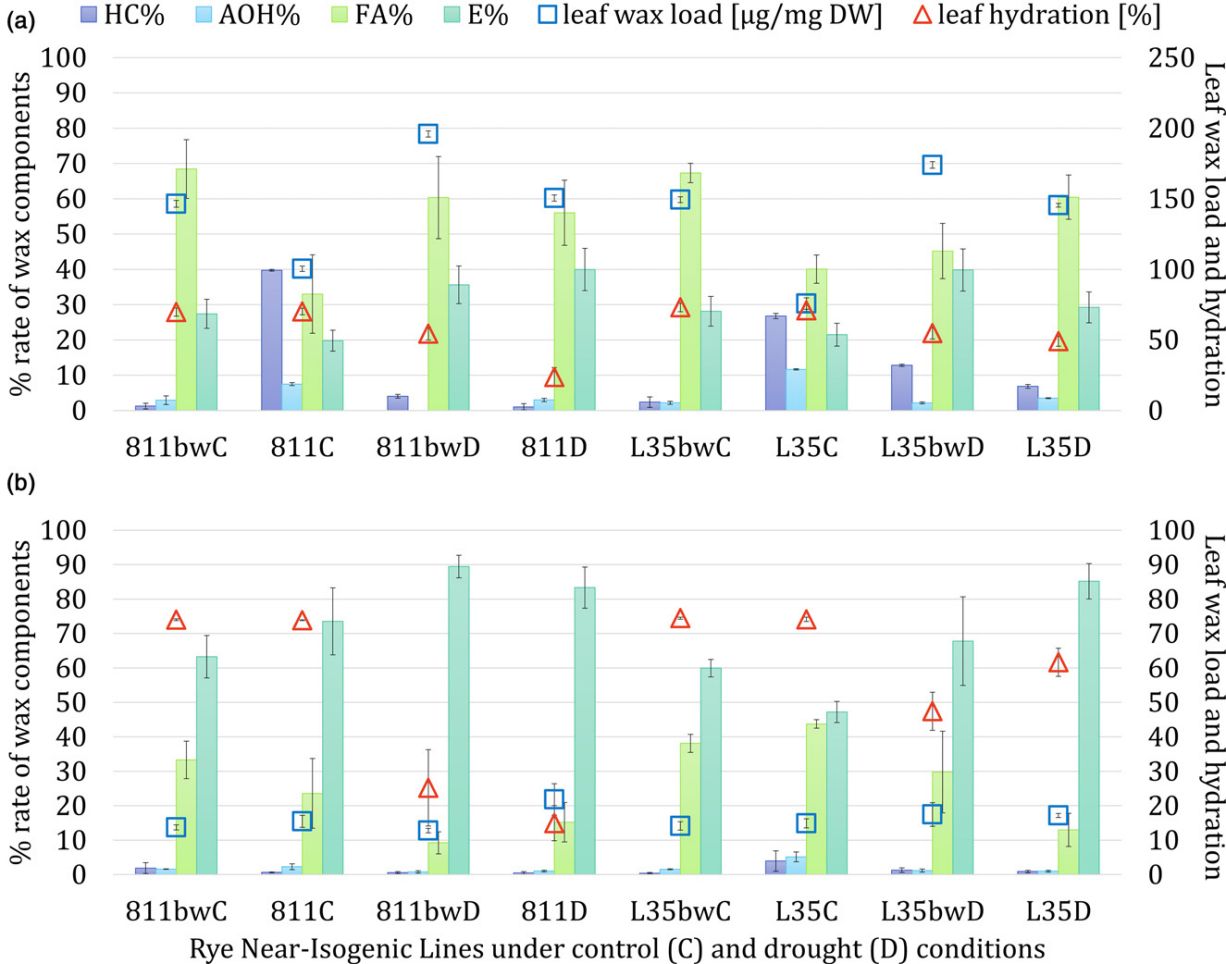
Rye leaf wax load (μg mg^−1^ DW) and leaf hydration (%) and percentage rate of rye leaf epicuticular wax components. Hydrocarbons (HC), primary alcohols (AOH), fatty acids (FA), and esters (E) during (a) first (2015) and (b) second (2016) year of experiment under control conditions (C) and soil drought stress (D). No data on alcohol fraction of 811bwD in 2015 due to loss of sample. 2015, pooled biological sample, technical errors are shown in (a); 2016, *n* = 3, mean and SE were calculated from three pooled biological samples.

**Figure 4:**
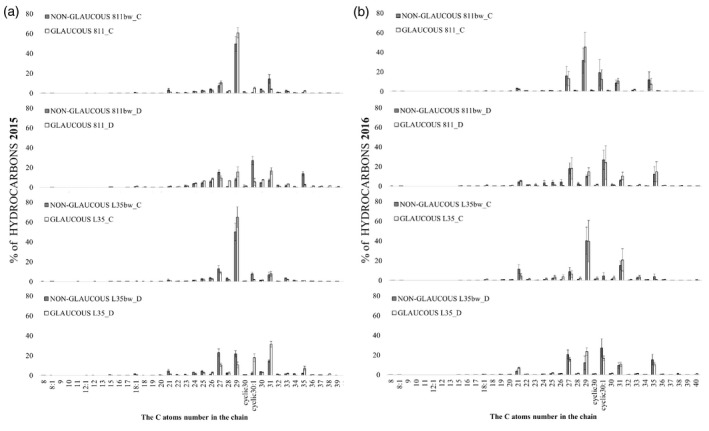
Gray bar indicate line with a recessive mutation disrupting the formation of the proper wax coating (non‐glaucous line) and white bar indicate typical wax line (glaucous line). Relative composition (%) of individual hydrocarbons in total hydrocarbons in leaf epicuticular wax of rye near‐isogenic lines under control conditions (C) and soil drought stress (D) during (a) first (2015) and (b) second (2016) year of the experiment. 2015, pooled biological sample technical errors are shown in (a); 2016, *n* = 3, mean and SE were calculated from three pooled biological samples.

**Figure 5:**
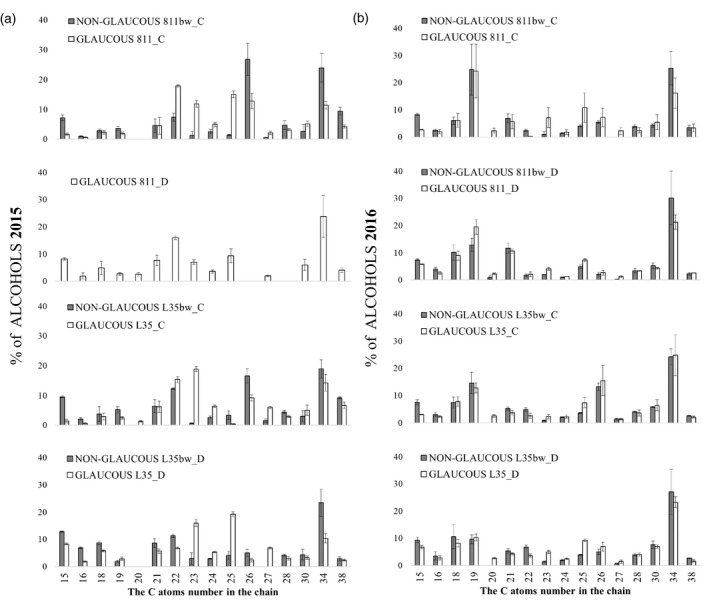
Gray bar indicate line with a recessive mutation disrupting the formation of the proper wax coating (non‐glaucous line) and white bar indicate typical wax line (glaucous line). Relative composition (%) of individual aliphatic alcohols in total alcohols in leaf epicuticular wax of rye near‐isogenic lines under control conditions (C) and soil drought stress (D) during (a) first (2015) and (b) second (2016) year of the experiment. No data on alcohol fraction of non‐glaucous 811bw_D in 2015 due to loss of sample. 2015, pooled biological sample, technical errors are shown in (a); 2016, *n* = 3; mean and SE were calculated from three pooled biological samples.

**Figure 6:**
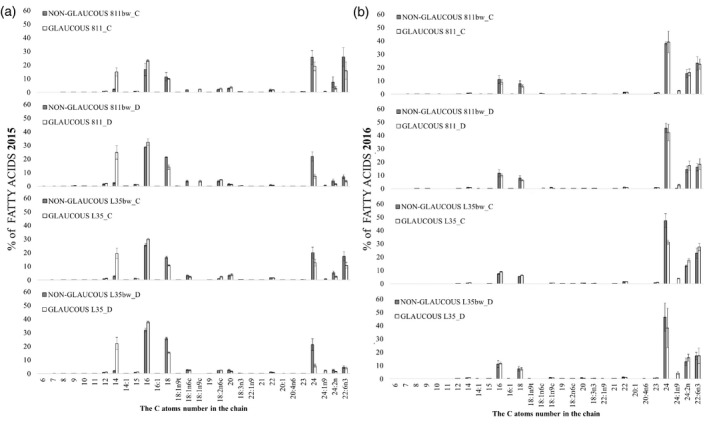
Gray bar indicate line with a recessive mutation disrupting the formation of the proper wax coating (non‐glaucous line) and white bar indicate typical wax line (glaucous line). Relative composition (%) of individual fatty acids in total fatty acids in leaf epicuticular wax of rye near‐isogenic lines under control conditions (C) and soil drought stress (D) during (a) first (2015) and (b) second (2016) year of the experiment. 2015, pooled biological sample, technical errors are shown in (a); 2016, *n* = 3, mean and SE were calculated from three pooled biological samples.

**Figure 7:**
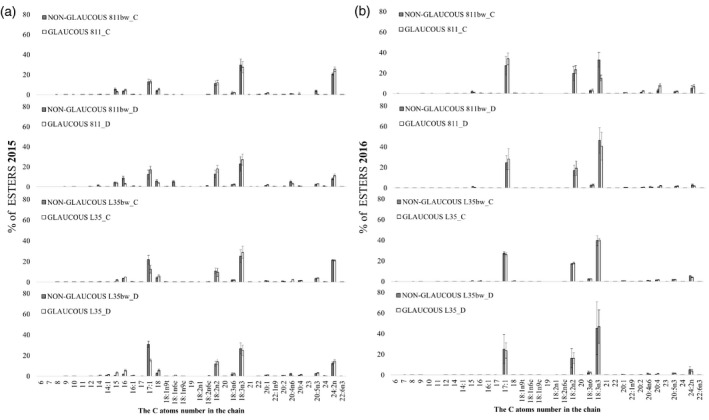
Gray bar indicate line with a recessive mutation disrupting the formation of the proper wax coating (non‐glaucous line) and white bar indicate typical wax line (glaucous line). Relative composition (%) of esters in total esters in leaf epicuticular wax of rye near‐isogenic lines under control conditions (C) and soil drought stress (D) during (a) first (2015) and (b) second (2016) year of the experiment. 2015, pooled biological sample, technical errors are shown in (a); 2016, *n* = 3, mean and SE was calculated from three pooled biological samples.

During the drought of 2015, hydration of non‐glaucous 811bw leaves (54.40%) was more than twice as high compared with the glaucous line 811 (23.69%); in the second tested pair, the leaves of the non‐glaucous line L35bw were more hydrated than the leaves of the glaucous line L35 only by approximately 5.70% (Figure 3a). During the drought of 2016, the non‐glaucous line 811bw again had better hydrated leaves than the glaucous line, but the difference was smaller than in 2015 and amounted to 10.29% (Figure 3b). In L35/L35bw pair, it was the glaucous line L35 that was characterized by 14.22% higher leaf hydration than its non‐glaucous counterpart, L35bw (Figure 3b). In the drought period of 2015, the non‐glaucous lines 811bw and L35bw had a higher leaf wax weight by 30% and 20%, respectively (Figure 3a). In the following year, under drought conditions, the non‐glaucous line 811bw was characterized by a lower weight of epicuticular wax than the glaucous line 811 by 41.44% (Figure 3b). These differences for the L35/L35bw NIL pair were much smaller under both treatments and ranged from 2% to 5% (Figure 3b). The glaucous and non‐glaucous lines of rye also differed in the content and percentage of individual wax fractions in leaf epicuticular wax; however, the differences were higher in 2015 (Figure 3). During the drought of 2015, the wax layer was dominated by the FA fraction (45–60% of the total fraction) in all rye lines, but they were higher in the glaucous lines 811 and L35 compared with the control value, while they were lower in the non‐glaucous lines (Figure 3a). The FA fraction was 15% higher in the glaucous line L35 compared with its non‐glaucous counterpart line L35bw (Figure 3a). The ester fraction (29–40%) was also significant, and its percentage increased during drought in all lines, while there was a clear 11% difference only in the pair of lines L35 and L35bw in favour of the non‐glaucous L35bw line (Figure 3a). The differences in the pair of lines 811 and 811bw were not so pronounced for FA and ester fractions (Figure 3a). In addition, a clear decrease in the percentage of hydrocarbons was observed in the glaucous lines, as opposed to the non‐glaucous lines 811bw and L35bw, where water deficits caused an increase in the proportion of hydrocarbons (Figure 3a). As in the previous year, the drought in 2016 resulted in a higher percentage of the ester fraction, but it was much more noticeable in 2016, as esters accounted for 86–91% of the analysed wax fractions (Figure 3b). The proportion of esters increased by 8–26% in both non‐glaucous lines 811bw and L35bw due to water deficits, while this increase in the glaucous line L35, wax approximately 40% (but the content was the lowest), and 9% increase in the glaucous line 811 (Figure 3b). In contrast to the previous year, differences between the glaucous and non‐glaucous lines in terms of hydrocarbons and primary alcohol fractions percentage rates were very low (about ≤4%) during drought conditions of 2016 (Figure 3b). Wax composition varied between individual years of the experiment both in terms of total hydrocarbons, fatty alcohols, FAs, and esters, as well as individual compounds of the collected fractions (Figures 3, 4, 5, 6, 7).

### Wax composition: hydrocarbon fraction

Regardless of the line, treatment, and year, the hydrocarbon fraction was dominated by odd‐chain alkanes (C27, C29, C31, C35) and cycloalkene C30:1 (squalene). More importantly, the glaucous and non‐glaucous NILs differed in the percentage of individual hydrocarbons (Figure 4). During the 2015 drought, the glaucous 811 and non‐glaucous L35bw lines showed a higher percentage of all odd‐chain compounds than lines 811bw and L35, by 6% and 9% respectively (Figure 4a). Both glaucous lines had a 9% higher percentage of these compounds in the following year than their non‐glaucous counterparts (Figure 4b). It is worth noting, however, that both glaucous and non‐glaucous lines had a reduced level of alkanes with odd chains in favour of even‐chain hydrocarbons during droughts in both years, mainly due to a higher percentage of cycloalkene C30:1 (Figure 4). During the drought of 2015, cyclic C30:1 dominated in the non‐glaucous line 811bw (27%) and glaucous line L35 (32%) (Figure 4a). Such observations in the following year were made in the non‐glaucous lines 811bw (27%) and L35bw (27%) and glaucous line 811 (24%) (Figure 4b).

### Wax composition: primary alcohol fraction

The glaucous and non‐glaucous lines showed different profiles of primary alcohols in wax under drought conditions, and the greatest differences were related to the proportion of C19, C22, C23, C25, C27, and C34 alcohols, which were repeated in both years, but they were smaller during the drought of 2016 compared with 2015 (Figure 5). Owing to the loss of alcohol fraction sample of the line 811bw from the 2015 drought, NIL 811/811bw could not be compared in that year in this regard (Figure 5a). In both years during water scarcity, the glaucous NIL L35 demonstrated a higher percentage of C23 (by 4–13%) and C25 alcohol (5–15%) than the non‐glaucous NIL, while the latter had a significantly higher percentage of C34 (by 3–13%) and C22 (3–5%) alcohol (Figure 5). Greater differences in the wax fatty alcohol profile in both NILs occurred in the first year, when a higher *T*_max_ occurred during drought (Figure 5a). During the drought of 2015, C27 alcohol (7% of the fraction) was detected in glaucous line L35, while it was not found in the fraction of non‐glaucous NIL L35bw; this compound was present in both control lines and constituted a low proportion in the alcohol profile in the following year (Figure 5). During the 2016 water deficit, the percentage of C19 in the alcohol fraction of the glaucous line 811 was 7% higher than in the non‐glaucous NIL, and similarly to the NIL L35, a higher percentage of C25 and C23 alcohols (2–3%) was determined (Figure 5b).

### Wax composition: FA fraction

The FA profile in rye leaf wax differed between the years (Figure 6). The fraction in 2015 in the glaucous lines was characterized by a high proportion of C14:0 and C16:0 short‐chain FAs, while C24:0 long‐chain FA was mostly found in the fraction from the non‐glaucous lines (Figure 6a). Contrary to the first year, both glaucous and non‐glaucous lines were dominated by C22–C24 long‐chain FAs in 2016 (Figure 6b). FA fractions in the epicuticular wax of rye NILs were dominated by palmitic acid (C16), octadecanoic acid (C18), lignoceric acid (C24), tetracosadienoic acid (C24:2n), and docosahexaenoic acid (C22:6n3c); the latter two accounted for a significant proportion of the FA fraction during the 2016 drought (Figure 6). The percentage of the above FAs differed between the years and glaucous and non‐glaucous lines (Figure 6). Only wax of the glaucous lines collected in 2015 was rich (15–25%) in myristic acid (C14:0), regardless of the treatment (Figure 6a). Thus, during the drought of 2015, the lines 811 and L35 contained a significantly higher percentage of C14 (20–22%), and, to a lesser extent, C16 (4–7%), whereas the non‐glaucous lines 811bw and L35bw were characterized by a higher percentage of C18 (7–10%) and C24 (15%) (Figure 6a). During the drought of the following year, clearer differences in the percentage composition of the FA fraction were observed in the L35/L35bw pair, while the differences in the 811/811bw NIL pair were low and reached a maximum of 3% (Figure 6b). Wax of the non‐glaucous line L35bw contained a higher percentage of C24 (8%) (Figure 6b). The glaucous lines in 2016 had a low percentage of C24:1n9 FA in the FA fraction, which was not observed in the non‐glaucous lines (Figure 6b).

### Wax composition: ester fraction

The percentage composition of the ester fraction differed between years; it was more variable also between NIL pairs in the first year when a higher *T*_max_ was recorded during the 3‐week drought (Figure 7). The wax fraction collected during the first year from drought‐treated plants was characterized by the highest proportion of C17:1 (12–31%), C18:2n2 (12–18%), C18:3n3 (23–27%), and C24:2n (8–15%) esters (Figure 7a). The latter was somewhat less abundant in wax collected in 2016 (2–5%), and 18:3n3 was the dominant ester during the 2016 drought, reaching even 47% (Figure 7b). The differences between the glaucous and non‐glaucous lines during water deficit, regardless of the year, mostly reached a maximum of 6% (Figure 7), except for the percentage of C17:1 ester, which was 16% higher in the 2015 drought in the non‐glaucous L35bw line compared with glaucous NIL (Figure 7a). Under the drought, the wax ester fraction in the glaucous line 811 had a higher proportion of C17:1 ester (5%) in both years, and for the non‐glaucous line, these were C16 (by 6%) and C18:1n6c esters (5%) in 2015 (Figure 7a) and C18:3n3 (by 6%) in 2016 (Figure 7b). The differences in the L35/L35bw pair were minor (maximum 2%) compared with the pair 811/811bw (Figure 7b) during drought of 2016.

### ChlA fluorescence (FC) parameters

Differences were found between the glaucous and non‐glaucous lines in NIL pairs with regard to ChlA fluorescence measurements on the flag leaf (Table 4). The non‐glaucous line 811bw was generally characterized by a better photochemical efficiency of the photosystem (PS) II leaf system (P.I.) and related parameters (ABS/CSm, ETo/CSm, TRo/CSm, RC/CSm), and lost the least energy due to heat dissipation (DIo/CSm) during drought (Table 4). Soil water deficiency caused a decrease in FC parameters almost to zero during the drought of 2015 and no valid FC measurements were made during the following year in the glaucous line 811 (Table 4). Its non‐glaucous equivalent, NIL 811bw, was characterized by a reduced value of the absorbed energy flux (ABS/CSm) and number of reaction centres (RC/CSm) during the drought in 2015, while all FC parameters decreased by 39–66% in the following year (Table 4). The lines in the second NIL pair, glaucous L35 and non‐glaucous L35bw, did not differ significantly in the value of FC parameters because of the drought in 2015 (Table 4). The effect of the drought also did not reduce FC values (Table 4). In the following year, the non‐glaucous line L35bw had significantly lower values (up to 32%) compared with the glaucous line, because of a stronger drought‐induced decrease in FC parameters in the non‐glaucous line than in the glaucous line (Table 4).

**Table 4:** Mean ± SE of chlorophyll *a* fluorescence (FC) parameters (PI, ABS/CSm, TRo/CSm, ETo/CSm, RC/CSm, DIo/CSm) and photosynthetic pigments content [chlorophyll *a* (ChlA), *b* (ChlB), ratio of chlorophylls *a* and *b* (Ratio ChlA/ChlB), total chlorophyll (TChl) and carotenoids (Car)] of rye near‐isogenic lines (NILs) under control (C) and drought (D) conditions during two study years (2015–2016) and percentage comparison of the non‐glaucous(N‐G) and glaucous lines (G) (N‐G/G%, glaucous line as a reference)

NILs		N‐G 811bw_C	G 811_C	N‐G/G (%)	N‐G L35bw_C	G L35_C	N‐G/G (%)	N‐G 811bw_D	G 811_D	N‐G /G (%)	N‐G L35bw_D	G L35_D	N‐G/G (%)
PI	2015	5.47 ± 0.63 ab	6.16 ± 0.73 ab	89	6.99 ± 0.91 a	6.15 ± 0.78 ab	114	3.91 ± 0.77 b	0.003 ± 0.002c	130375	5.06 ± 0.69 ab	4.29 ± 0.63 b	118
	2016	4.48 ± 0.28 ab	5.82 ± 0.42 a	77	3.53 ± 0.4 bc	4.06 ± 0.19 bc	87	1.84 ± 1.8 d	0 ± 0 e	ND	1.69 ± 0.52 d	2.7 ± 0.32 cd	62
ABS/CSm	2015	1202 ± 32.07 a	1268.4 ± 39.02 a	95	1128.8 ± 45.33 ab	1087 ± 43.8 ab	104	969.25 ± 85 b	301.5 ± 1.5 c	321	1198.71 ± 65.05 a	1123.71 ± 80.86 ab	107
	2016	1060.4 ± 19.26 a	1122.75 ± 19.71 a	94	1046.18 ± 26.73 a	1110 ± 20.1 a	94	439 ± 288 c	0 ± 0 d	ND	692.89 ± 116.7 b	924.07 ± 64.99 a	75
TRo/CSm	2015	984.25 ± 32.95 ab	1041.2 ± 40.42 a	95	918.2 ± 47.48 ab	876 ± 44.47 ab	105	756.75 ± 79.29 b	27.5 ± 4.5 c	2752	981.71 ± 59.68 ab	901 ± 84.8 ab	109
	2016	866.1 ± 18.44 a	923.67 ± 18.27 a	94	832.09 ± 25.96 a	891.92 ± 18.53 a	93	320 ± 272 c	0 ± 0 d	ND	512 ± 110.33 b	735.36 ± 57.06 a	70^*^
ETo/CSm	2015	676 ± 26.76 ab	739 ± 40.15 a	91	656 ± 45.88 ab	618.6 ± 36.13 ab	106	518.5 ± 74.59 b	8.5 ± 2.5 c	6100	660.86 ± 47.09 ab	598 ± 65.18 ab	111
	2016	557.8 ± 17.93 ab	636 ± 20 a	88	534.73 ± 26.63 ab	592 ± 16.16 a	90	190.5 ± 172.5 c	0 ± 0 d	ND	303 ± 74.79 c	444.93 ± 38.65 b	68
RC/CSm	2015	654.77 ± 39.85 a	678.01 ± 37.78 a	97	705.73 ± 44.48 a	655.68 ± 46.29 a	108	483.4 ± 38.69 b	16.89 ± 7.11 c	2862	619.79 ± 46.53 ab	579.53 ± 50.49 ab	107
	2016	582.34 ± 15.29 a	621.17 ± 19.19 a	94	506.32 ± 24.64 ab	553.79 ± 15.63 a	91	201.65 ± 179.15 c	0 ± 0 d	ND	282.36 ± 63.95 c	417.76 ± 41.51 b	68
DIo/CSm	2015	217.75 ± 3.99 b	227.2 ± 2.52 b	96	210.6 ± 3.31 b	211 ± 5.21 b	100	212.5 ± 6.02 b	274 ± 3 a	78	217 ± 9.04 b	222.71 ± 5.41 b	97
	2016	194.3 ± 1.99 bc	199.08 ± 2.29 abc	98	214.09 ± 2.61 ab	218.08 ± 3.45 a	98	119 ± 16 d	0 ± 0 e	ND	180.89 ± 7.67 c	188.71 ± 9.31 c	96
ChlA (µg/mg DW)	2015	11.42 ± 0.87 a	10.28 ± 0.11 b	111	9.04 ± 0.21 c	8.51 ± 0.29 cd	106	7.53 ± 0.42 d	1.94 ± 0.03 f	389	3.91 ± 0.07 e	2.79 ± 0.08 f	140
	2016	4.76 ± 0.09 b	5.59 ± 0.05 a	85	3.46 ± 0.03 d	3.9 ± 0.06 c	89	1.71 ± 0.15 g	0.75 ± 0.01h	227	1.96 ± 0.02 f	2.35 ± 0.03 e	83
ChlB (µg/mg DW)	2015	3.11 ± 0.25 a	2.91 ± 0.03 a	107	2.34 ± 0.07 b	2.22 ± 0.07 bc	106	1.95 ± 0.1 c	0.78 ± 0.01 de	249	1.02 ± 0.02 d	0.71 ± 0.02 e	144
	2016	1.11 ± 0.08 bc	1.3 ± 0.03 ab	86	0.87 ± 0.01 bcd	1.04 ± 0.01 bc	83	1.74 ± 0.51 a	0.39 ± 0.02 d	442	0.69 ± 0.03 bcd	0.63 ± 0.01 cd	111
Ratio ChlA/ChlB	2015	3.67 ± 0.04 c	3.53 ± 0.02 d	104	3.86 ± 0.03 b	3.83 ± 0 b	101	3.85 ± 0.04 b	2.47 ± 0.005 e	156	3.82 ± 0.02 b	3.94 ± 0.03 a	97
	2016	4.34 ± 0.22 a	4.32 ± 0.1 ab	100	3.97 ± 0.03 abc	3.73 ± 0,04 c	106	1.41 ± 0.43 e	1.93 ± 0.09 e	73	2.84 ± 0.11 d	3.76 ± 0.01 bc	76
TChl (µg/mg DW)	2015	14.53 ± 1.12 a	13.19 ± 0.13 a	110	11.39 ± 0.28 b	10.74 ± 0.36 bc	106	9.48 ± 0.51 c	2.72 ± 0.04 e	349	4.93 ± 0.09 d	3.5 ± 0.1 e	141
	2016	5.88 ± 0.16 b	6.89 ± 0.06 a	85	4.33 ± 0.03 c	4.94 ± 0.07 c	88	3.44 ± 0.65 d	1.14 ± 0.03 f	301	2.65 ± 0.04 e	2.98 ± 0.04 de	89
Car (µg/mg DW)	2015	3.13 ± 1.12 a	2.82 ± 0.13 b	111	2.45 ± 0.28 c	2.24 ± 0.36 c	109	2.22 ± 0.51 c	0.57 ± 0.04 e	391	1.25 ± 0.09 d	0.98 ± 0.1 d	128
	2016	1.44 ± 0.02 b	1.7 ± 0.02 a	85	1.1 ± 0.01 c	1.09 ± 0.02 c	101	0.39 ± 0.08 f	0.4 ± 0.01 f	99	0.65 ± 0.01 e	0.84 ± 0.01 d	78

Different letters indicate differences between NILs (each year separately) according to the Duncan test (*P* ≤ 0.05). ABS/CSm, light energy absorption; DIo/CSm, amount of energy dissipated from PSII; ETo/CSm, amount of energy used for electron transport; FC parameters: PI, overall performance index of photosystem II (PSII) photochemistry; ND, no data, lack of comparison because of zero values obtained by glaucous line 811 for chosen parameters in 2015 (PI, ABS/CSm, TRo/CSm, ETo/CSm, RC/CSm, DIo/CSm); RC/CSm, number of active reaction centres; TRo/CSm, amount of excitation energy trapped in PSII reaction centres.

### Photosynthetic pigment content

Photosynthetic pigment content (PPC) levels varied between the glaucous and non‐glaucous lines in both NIL pairs in control and scarce water conditions (Table 4). Under the drought of 2015, chlorophyll content (ChlA, ChlB, TChl) in the non‐glaucous lines were higher than in the glaucous ones, up to three times in the line 811bw and 40–45% higher in the non‐glaucous line L35bw (Table 4). Significantly higher more Car content (4‐fold) was detected in the non‐glaucous line 811bw compared with the glaucous line 811 during the drought of 2015, while no significant differences were observed in the NIL pair L35/L35bw (Table 4). The glaucous and non‐glaucous lines differed from each other in the drought of 2016 in terms of PPC; however, the direction of changes and their magnitude was different for the analysed pairs (Table 4). The non‐glaucous line 811bw was characterized by up to 4‐fold higher content of chlorophylls, without a significantly different Car value (Table 4). In turn, the non‐glaucous L35bw line had significantly lower levels of ChlA and Car by 17% and 22%, respectively (Table 4). In 2015, under both treatments, the non‐glaucous 811bw line was characterized by a significantly higher ChlA/B ratio (4–56%) than the glaucous line, but no differences were found in 2016 (Table 4). A significantly higher ChlA/B ratio (by 3–25%) was found for the glaucous line L35 during drought in both years (Table 4). It is worth noting that for both the glaucous and non‐glaucous lines, the drought resulted in a 23–87% decrease in PPC levels in the flag leaf (Table 4). Exceptionally, during the drought of 2016, ChlB level dropped significantly (70%) in comparison with other PPC only for the glaucous line 811, whereas its non‐glaucous counterpart showed an increased ChlB level by 56% (Table 4). Differences between the glaucous and non‐glaucous lines were also visible in the level of individually measured carotenoids (Table 5). The non‐glaucous line 811bw was characterized by a significantly higher content of Zea, β‐car, Lut, and Viol than the glaucous line 811 in 2015 and 2016 (2–7‐fold and 52–58%, respectively) (Table 5). Significantly stronger differences were recorded for 2015 when a higher *T*_max_ occurred (Figure 1). Differences were also observed in the second pair; the non‐glaucous NIL L35bw had a significantly higher value of Lut, Zea, and β‐car compared with the glaucous NIL L35 under the drought of 2015 (by 11–21%), while drought conditions in 2016 caused significant differences in favour of the glaucous line L35 (by 17–19%) (Table 5). Both pairs of glaucous and non‐glaucous NILs had a reduced content of Viol, Lut, and β‐car under drought conditions in both years; however, Zea content increased during the drought of 2015 in the non‐glaucous NILs 811bw (by 68%), L35bw (23%), and glaucous NIL L35 (by 5%), while it decreased in glaucous NIL 811 by 74% (Table 5). The 811/811bw NIL pair showed a decreased Zea level (by 61–80%) in the following year during water scarcity conditions, while no significant differences were observed in the L35/L35bw NIL pair (Table 5).

**Table 5:** Mean values ± SE of total phenolic content (TPC), soluble sugar content (SSC), tocotrienols (α, γ, δ‐T3), tocopherols (α, β, γ, δ), carotenoids [vioxanthin (Viol), zeaxanthin (Zea), lutein (Lut), β‐carotene (β‐car)], and malondialdehyde (MDA) contents of rye near‐isogenic lines (NILs) under control (C) and drought (D) conditions during 2 years (2015–2016) of the experiment and percentage comparison of the non‐glaucous (N‐G) and glaucous lines (G) (N‐G/G%, glaucous line as a reference)

NILs		N‐G 811bw_C	G 811_C	N‐G/G (%)	N‐G L35bw_C	G L35_C	N‐G/G (%)	N‐G 811bw_D	G 811_D	N‐G/G (%)	N‐G L35bw_D	G L35_D	N‐G/G (%)
TPC (µg/mg DW)	2015	4.44 ± 0.28 a	3.45 ± 0.04 c	129	4.58 ± 0.11 a	4.41 ± 0.08 a	104	4.1 ± 0.28 ab	2.3 ± 0.02 d	178	4.28 ± 0.05 ab	3.81 ± 0.15 bc	112
	2016	5.04 ± 0.03 b	5.26 ± 0.04 a	96	4.77 ± 0.05 c	3.71 ± 0.07 f	129	3.6 ± 0.02 fg	3.49 ± 0.03 g	103	4.42 ± 0.05 d	4.03 ± 0.04 e	110
SSC (µg/mg DW)	2015	73.79 ± 4.66 b	55.44 ± 1.46 c	133	84.21 ± 1.89 a	78,87 ± 1.82 ab	107	44.43 ± 1.94 d	16.37 ± 0.6 f	271	39.25 ± 0.65 d	32.67 ± 1.68 e	120
	2016	113.82 ± 1.71 c	128.82 ± 1.13 a	88	118.13 ± 2.21 b	108.11 ± 1.9 d	109	34.18 ± 0.71 g	28.47 ± 0.91h	120	54.53 ± 0.4 f	80.41 ± 1.55 e	68
Viol (ng/mg DW)	2015	62.92 ± 3 c	93.99 ± 4.52 a	67	84.47 ± 1.36 b	65.67 ± 0.74 c	129	45.09 ± 2.31 d	22.47 ± 0.57 e	201	20.44 ± 0.7 e	18.38 ± 0.39 e	111
	2016	79.84 ± 4.26 b	98.93 ± 2.96 a	81	24.96 ± 1.78 c	25.61 ± 2.14 c	97	18.01 ± 0.46 d	11.59 ± 1.07 e	155	12.26 ± 0.89 de	16.75 ± 0.57 de	73
Lut (ng/mg DW)	2015	854.66 ± 4.89 b	923.79 ± 7.23 a	93	677.83 ± 5.49 d	627.83 ± 6.78 e	108	729.99 ± 3.66 c	218.57 ± 1.84 h	334	350.91 ± 1.92 f	290.67 ± 4.15 g	121
	2016	535.96 ± 8.74 b	603.97 ± 8.93 a	89	315.35 ± 4.6 c	316.85 ± 13.07 c	100	216.4 ± 1.5 e	142.15 ± 1.29 f	152	212.49 ± 2.42 e	255.73 ± 1.92 d	83
Zea (ng/mg DW)	2015	133.54 ± 2.07 b	116.88 ± 0.5 de	114	112.16 ± 0.95 e	118.52 ± 1.51 d	95	224.35 ± 2.89 a	30.47 ± 1.19 f	736	137.57 ± 0.84 b	124.11 ± 2.19 c	111
	2016	78.72 ± 2.49 b	82.8 ± 5.71 b	95	94.39 ± 1.82 a	95.64 ± 4.2 a	99	64.29 ± 0.47 c	38.38 ± 0.47 d	168	93.26 ± 1.46 a	100.5 ± 1.03 a	93
β‐car (ng/mg DW)	2015	2703.02 ± 14.38 a	2763.26 ± 42.22 a	98	2267.76 ± 7.71 b	2114.83 ± 21.42 c	107	2076.11 ± 25.07 c	434.63 ± 2.11 f	478	1043.17 ± 6.47 d	857.4 ± 10.75 e	122
	2016	1551.31 ± 34 b	1809.81 ± 15.05 a	86	992.38 ± 19.59 c	1013.89 ± 39.05 c	98	610.96 ± 3.66 e	370.73 ± 2.32 f	165	661.11 ± 4.23 e	813.3 ± 1.69 d	81
α‐T3 (ng/mg DW)	2015	4.51 ± 0.3 d	3.41 ± 0.15 e	132	1.73 ± 0.19 f	0.6 ± 0.09 g	290	11.95 ± 0.26 a	10.36 ± 0.11 b	115	6.13 ± 0.3 c	4.6 ± 0.1 d	133
	2016	4.74 ± 0.31 a	4.07 ± 0.38 a	116	1.35 ± 0.04 d	1.54 ± 0.18 d	88	4.64 ± 0.17 a	3.42 ± 0.16 b	136	2.2 ± 0.22 c	2.65 ± 0.04 c	83
γ‐T3 (ng/mg DW)	2015	1.35 ± 0.15 a	1.13 ± 0.11 ab	120	0.98 ± 0.03 b	1.04 ± 0.1 ab	94	1.07 ± 0.02 ab	0.09 ± 0.01 c	1190	1.21 ± 0.19 ab	0.95 ± 0.13 b	127
	2016	0.52 ± 0 b	0.25 ± 0.02 c	210	0.78 ± 0.07 ab	0.86 ± 0.13 a	91	0.66 ± 0.01 ab	0.64 ± 0.05 ab	103	0.85 ± 0.14 a	0.76 ± 0.06 ab	112
δ‐T3 (ng/mg DW)	2015	0.59 ± 0.01 f	0.64 ± 0.03 ef	93	0.94 ± 0.02 c	0,67 ± 0 e	140	0.67 ± 0.04 e	1.68 ± 0.03 a	40	0.78 ± 0.02 d	1.34 ± 0.01 b	58
	2016	0.05 ± 0.03 b	0 ± 0 b	ND	0.03 ± 0.02 b	0.26 ± 0.04 a	11	0.07 ± 0.02 b	0.04 ± 0.01 b	158	0.29 ± 0.03 a	0.23 ± 0.01 a	124
α‐T (ng/mg DW)	2015	185.03 ± 25.57 b	143.7 ± 13.66 c	129	78.59 ± 5.86 e	86.79 ± 3.22 de	91	223.74 ± 5.14 a	109.71 ± 3.73 cde	204	120.27 ± 13.63 cd	122.25 ± 8.32 cd	98
	2016	283.39 ± 97.61 a	196.22 ± 12.23 b	144	161.25 ± 12.25 bc	139.6 ± 11.33 bc	116	113.98 ± 2.05 bc	80.63 ± 6.02 c	141	190.72 ± 31.81 b	197.3 ± 6.83 b	97
β‐T (ng/mg DW)	2015	1.99 ± 0.09 cd	0.87 ± 0.06 e	230	2.02 ± 0.01 cd	1.55 ± 0.06 d	131	2.09 ± 0.38 bc	0.25 ± 0.1 f	840	2.54 ± 0.11 ab	2.57 ± 0.05 a	99
	2016	1.34 ± 0.03 c	1.25 ± 0.04 c	107	1.42 ± 0.05 bc	1.08 ± 0.09 c	131	2.1 ± 0.19 a	2.33 ± 0.13 a	90	2.17 ± 0.09 a	1.69 ± 0.06 b	128
γ‐T (ng/mg DW)	2015	5.81 ± 0.12 c	2.73 ± 0.09 d	213	2.78 ± 0.03 d	2.68 ± 0.07 d	103	8.07 ± 0.34 b	8.92 ± 0.43 a	91	7.62 ± 0.07 b	8.8 ± 0.19 a	87
	2016	4.53 ± 0.2 de	3.65 ± 0.09 e	124	8.57 ± 0.65 c	5.63 ± 0.27 d	152	14.85 ± 0.17 b	15.35 ± 0.51 b	97	20.64 ± 0.51 a	14.48 ± 0.07 b	143
δ‐T (ng/mg DW)	2015	0.11 ± 0.01 d	0 ± 0 e	ND	0.02 ± 0 e	0.03 ± 0 e	54	0.23 ± 0.02 c	0.65 ± 0.05 a	36	0.36 ± 0.01 b	0.35 ± 0.04 b	102
	2016	0.21 ± 0.04 e	0.17 ± 0.02 e	124	0.33 ± 0.02 d	0.2 ± 0.02 e	165	0.78 ± 0.03 b	0.99 ± 0.02 a	80	0.48 ± 0.03 c	0.34 ± 0.02 d	139
MDA (µg/mg DW)	2015	0.09 ± 0.01 b	0.09 ± 0.01 b	104	0.07 ± 0.003 b	0.08 ± 0.005 b	87	0.09 ± 0.001 b	0.14 ± 0.003 a	62	0.08 ± 0.003 b	0.09 ± 0.004 b	96
	2016	0.11 ± 0.001 b	0.11 ± 0.002 b	94	0.1 ± 0.002 b	0.08 ± 0.015 c	133	0.15 ± 0.006 a	0.17 ± 0.009 a	90	0.1 ± 0.005 bc	0.11 ± 0.006 b	92

Different letters indicate differences between NILs (each year separately) according to the Duncan test (*P* ≤ 0.05). ND, no data, lack of comparison because of zero values obtained by glaucous line 811 for chosen parameters in 2015 (δ‐T3) and in 2016 (δ‐T).

### Tocochromanol and MDA content

Differences between NIL pairs were found with regard to tocochromanol (tocopherols α‐, β‐, γ‐, δ‐T, and tocotrienols α, γ‐, and δ‐T3) level in both years and treatments (Table 5). During the 2015 drought, when a significantly higher *T*_max_ was also recorded (Figure 1), the non‐glaucous 811bw line differed very clearly from the glaucous line 811 in its γ‐T3 and β‐T content, which was up to 12‐fold higher, and had a 2‐fold higher α‐T content, but lower content of δ‐T3 (by 60%) and δ‐T (by 64%) (Table 5). The following year did not bring such clear differences between the lines in this pair; under drought, the 811bw line had a 36% higher level of α‐T3, while δ‐T level was again significantly higher in the glaucous line 811, but this time by 20% (Table 5). Under the drought in 2015, the leaves of the non‐glaucous line L35bw had a significantly higher content of α‐T3 (by 33%), but lower content of δ‐T3 and γ‐T (by 42% and 13%, respectively) (Table 5). During the drought in 2016, the leaves of the non‐glaucous L35bw line had a significantly higher content of tocopherols β‐T, δ‐T, and γ‐T (by 28–43%) (Table 5). Tocochromanol levels observed under drought varied in both years, most of them were significantly increased because of water deficit and these increases were not equal in the glaucous and non‐glaucous lines (Table 5). The highest increase among the most abundant tocochromanols in rye leaves was recorded for γ‐T, which, increased three to four times in the glaucous lines and two to three times in the non‐glaucous lines due to water deficiency (Table 5).

The MDA value was significantly higher in the glaucous line 811 only during the 2015 drought compared with the non‐glaucous line 811bw (Table 5). There were no significant differences recorded between lines in pairs during the drought in 2016 (Table 5). Surprisingly, in the first year, when the maximum air temperature was higher (Figure 1), a significantly increased MDA level under drought was observed only in the glaucous 811 line (by 61%), while during water deficit in the following year, it significantly increased (by 40–49%) in all lines except the non‐glaucous NIL L35bw, in which MDA did not change significantly (Table 5).

### TPC

The results obtained during both experiments revealed significant differences between the glaucous and non‐glaucous lines among the tested pairs of NILs under both treatments; however, under water‐deficit conditions, the lines in the 811/811bw pair differed significantly in 2016, and in the L35/L35bw pair in 2015 (Table 5). The differences indicated significantly higher amounts of TPC in the non‐glaucous lines 811bw and L35bw (by 78% and 10%, respectively) (Table 5). During both years of the experiment, a decrease in TPC was induced by water shortage; however, the differences were not significant for all lines and the decline reached a maximum of 33% (Table 5). Unlike other lines, the glaucous line L35 significantly increased TPC (8%), but only under water shortage in 2016 (Table 5). The glaucous line 811 showed the highest significant TPC decrease after a 3‐week drought during both years. In addition, the lines of the 811/811bw NIL pair differed from the lines L35 and L35bw in a greater decrease in TPC levels due to drought (Table 5).

### SSC

The glaucous and non‐glaucous lines differed significantly within NIL pairs under both conditions (Table 5). In the first year of the experiment under water scarcity, when higher maximum temperatures occurred (Figure 1), the non‐glaucous lines showed significantly higher SSC than their glaucous equivalents, as the non‐glaucous line 811bw had almost three times more soluble sugars than the glaucous one, while smaller differences occurred between NILs L35bw and L35 (20% in favour of non‐glaucous NIL) (Table 5). There were some changes in the second year in comparison with 2015; the non‐glaucous line 811bw accumulated higher SSC under drought stress than glaucous 811 (20%), whereas the opposite results were obtained for the non‐glaucous line L35bw in the form of lower SSC accumulation (32%) (Table 5). Drought significantly decreased (25–77%) the level of SSC in all lines during the first and the second year of the experiment (Table 5). The highest decrease was recorded for the glaucous line 811 (both years), and the lowest for the non‐glaucous line 811bw (2015) and non‐glaucous line L35bw (2016) (Table 5). Differences between pairs were observed during both experiments, lines 811 and 811bw showed a higher decrease in SSC (39–77%) than lines L35 and L35bw (25–58%) under drought stress (Table 5).

### Yield components, above‐ground plant biomass, and plant height

The differences between glaucous and non‐glaucous lines were observed under both conditions; however, the direction of changes differed (Table 6). There were no significant differences in plant height between the lines in NIL pairs under optimum soil moisture; however, because of a 3‐week drought in 2015, the non‐glaucous line 811bw was 19% taller than the glaucous one (Table 6). The glaucous line 811 did not produce any seeds after the water shortage in 2015, as opposed to the second year of the experiment (Table 6); however; under the 2016 drought, the non‐glaucous 811bw was characterized by a significantly higher (70%) TGW (Table 6). Under control conditions in 2015, the non‐glaucous line L35bw produced fewer and lighter seeds than the glaucous L35, but no significant differences in yield were noticed during the water shortage (Table 6). Under the drought in 2016, seeds of the glaucous line L35 were 32% heavier than the non‐glaucous line L35bw seeds, and TGW was significantly lower (19%) in the non‐glaucous line L35bw (Table 6). There were no significant differences in biomass in the pairs of glaucous and non‐glaucous NILs under water deficit during both experimental years (Table 6). Both study years revealed differences between the pairs of NILs (Table 6). Under the drought in 2015, when the maximum temperature was significantly higher (Figure 1), both NIL pairs exhibited significantly decreased plant height (25–46%), GN (59–100%), GW (68–100%), and TGW (20–100%) (Table 6), while only the NILs 811 and 811bw showed a decrease in those parameters under water shortage in 2016 (Table 6).

**Table 6:** Mean ± SE and the percentage of control (%C) of plant height with awns (PH), grain number (GN) and grain weight (GW) per plant, thousand grain weight (TGW), above‐ground biomass (B), and δ^13^C carbon isotope discrimination in rye near‐isogenic lines (NILs) under control (C) and drought (D) conditions during 2 years (2015–2016) of the experiment and percentage comparison of the non‐glaucous(N‐G) and glaucous lines (G) (N‐G/G%, glaucous line as a reference)

NILs		N‐G 811bw_C	G 811_C	N‐G/ G(%)	N‐G L35bw_C	G L35_C	N‐G/ G(%)	N‐G 811bw_D	G 811_D	N‐G/ G(%)	N‐G L35bw_D	G L35_D	N‐G/ G(%)
PH (cm)	2015	93.13 ± 3.77 a	97.14 ± 1.96 a	96	52.88 ± 3.5 c	59 ± 2.83 bc	90	62.07 ± 5.36 b	52.14 ± 2.01 c	119	39.64 ± 2.95 d	40.93 ± 1.69 d	97
	2016	106.02 ± 2.73 a	110.93 ± 1.41 a	96	47.65 ± 1.86 c	50.21 ± 2.16 c	95	97.21 ± 2.05 b	94.55 ± 1.87 b	103	47.2 ± 1.73 c	52.91 ± 1.41 c	89
GN	2015	233 ± 40 a	217 ± 25 a	107	79 ± 29 b	185 ± 16 a	43	90 ± 5 b	0 ± 0 c	ND	68 ± 19 b	75 ± 18 b	91
	2016	148 ± 10 b	185 ± 7 a	80	86 ± 7 c	80 ± 6 c	107	89 ± 9 c	99 ± 10 c	89	77 ± 3 c	93 ± 6 c	83
GW (g)	2015	4.17 ± 0.8 a	4.33 ± 0.56 a	96	1.49 ± 0.52 b	3.91 ± 0.34 a	38	1.03 ± 0.08 bc	0 ± 0 c	ND	1.04 ± 0.3 bc	1.23 ± 0.3 bc	85
	2016	2.75 ± 0.2 b	3.69 ± 0.15 a	75	1.72 ± 0.15 cd	1.86 ± 0.11 cd	92	0.83 ± 0.2 e	0.57 ± 0.17 e	144	1.4 ± 0.08 d	2.06 ± 0.1 c	68
TGW (g)	2015	17.61 ± 0.82 bc	19.89 ± 0.86 a	89	19.16 ± 0.3 ab	21.1 ± 0.22 a	91	11.48 ± 0.21 e	0 ± 0 f	ND	15.27 ± 0.16 d	15.75 ± 1.16 cd	97
	2016	18.64 ± 0.31 b	19.91 ± 0.25 b	94	19.84 ± 0.61 b	23.49 ± 0.67 a	84	8.07 ± 1.53 c	4.74 ± 1.14 d	170	18.2 ± 0.71 b	22.49 ± 0.64 a	81
B (g)	2015	8.82 ± 1.71 ab	10.83 ± 0.89 a	81	4.53 ± 1.2 de	7.85 ± 0.78 bc	58	4.88 ± 1.13 cde	3.51 ± 0.2 e	139	6.93 ± 1.28 bcd	6.02 ± 0.74 b–e	115
	2016	10.42 ± 0.69 b	12.52 ± 0.5 a	83	8.12 ± 0.54 c	6.76 ± 0.33 d	120	6.39 ± 0.14 d	6.39 ± 0.09 d	100	7.4 ± 0.23 cd	7.31 ± 0.29 cd	101
δ^13^C^b^	2015	–29.47 ± 0.03 e	–29.04 ± 0.06 d	101	–30.12 ± 0.01 g	–29.76 ± 0.03 f	101	–26.83 ± 0.08 c	–26.22 ± 0.03 a	102	–26.53 ± 0.03 b	–26.51 ± 0.07 b	100
	2016	–29.26 ± 0.05 d	–29.09 ± 0.05 c	101	–29.41 ± 0.02 e	–28.97 ± 0.02 b	102	–29.25 ± 0.02 d	–29.13 ± 0.06 c	100	–29.35 ± 0.02 de	–28.66 ± 0.04 a	102

Different letters indicate differences between NILs (each year separately) according to the Duncan test (*P* ≤ 0.05). ND, no data, lack of comparison because of zero values obtained by glaucous line 811 for chosen parameters in 2015 (GN, GW, TGW).

^a^ Percentage comparison [N‐G/G (%)] of δ13C should be interpreted inversely to the other cases (<100%, increase; >100%, decrease) due to negative values of parameter δ13C.

### ^13^C carbon isotope discrimination (δ^13^C)

The value of the δ^13^C index in leaves of rye NILs differed between the years, treatments, line pairs, and glaucous and non‐glaucous lines (Table 6). The glaucous lines were characterized by higher δ^13^C values than non‐glaucous lines regardless of the treatment and the year of the experiment, with the exception of NILs in the L35/L35bw pair in 2015 (Table 6). Higher *T*_max_ was recorded during the drought of 2015 in comparison with the second year, and both glaucous and non‐glaucous lines showed significantly higher δ^13^C values in relation to the control objects, while this result in the following year was observed only in the glaucous L35 line (Table 6).

## DISCUSSION

Differences in the measured parameters were observed between the two experiments conducted in 2015–2016 that could result from different air temperature conditions. *T*_max_ during the 3‐week period in 2015 was significantly higher when the plants were exposed to drought stress, particularly in the first week of drought, combined with a lower percentage of soil moisture than in the following year (Figure 1). Numerous studies have shown that different environmental factors affecting the plant cause its unique response to abiotic stresses (Balfagón et al., 2020; Khan et al., 2020; Tricker et al., 2018; Zandalinas et al., 2018) and influence the results, as shown in our research. Regarding differences in rye wax composition between the years, Sheperd and Griffiths (2006) reported that high temperature affected the composition of waxes; however, it was also dependent on irradiation and plant species. Our research concerned soil drought stress, while additional temperature analysis in the vegetation tunnel revealed that other environmental factors also affected rye plants. Thus, each year, a unique combination of environmental factors influenced the plants’ response to this combination of stresses, resulting in differences between wax component contents in the first and second year of the experiment.

Microstructural variability of the abaxial leaf blade wax layer of rye NILs reflected phenotypic differences between the glaucous and non‐glaucous lines. The presence of tubular wax crystals on the abaxial leaf blade observed in the glaucous NILs was also recorded in cereals such as wheat (Bi et al., 2017; Guo et al., 2016; Wang et al., 2015b) and barley (Hen‐Avivi et al., 2016). Their occurrence is associated with an increased content of β‐diketone compounds in wax (Barthlott et al., 1998; Bi et al., 2017; Lavergne et al., 2018; Su et al., 2020; Von Wettstein‐Knowles, 2016). The presence of β‐diketone tubules in wheat was shown to be associated with the occurrence of a glaucous drought‐resistant phenotype (Bi et al., 2016; Zhang et al., 2015). In control conditions, no variation in the type of wax crystals was observed in rye NILs. However, under drought, distinct platelet‐like crystalloids appeared on the abaxial leaf blade of the non‐glaucous line L35bw and remained undefined (Figure 2p). Studies concerning leaf wax morphology in conditions of water scarcity in wheat (Bi et al., 2017; Guo et al., 2016; Willick et al., 2018) demonstrated only changes in crystal density after drought treatment. However, a recent study on non‐glaucous and glaucous wheat cultivars performed by Su et al. (2020) showed a strong response of wax tubules to drought in both lines. A study by Sanjari et al. (2021) on leaf wax of drought‐tolerant (Kimia and KGS23) and drought‐susceptible (Sepideh) sorghum genotypes under drought stress, revealed both increased crystal density in Kimia and Sepideh genotypes and no changes in the tolerant KGS23 genotype. In the current study, there were no significant differences in densities of rye wax crystal because of drought, except for NIL L35bw (Figure 2). A study focusing on the morphology of Brussels sprout waxes (Baker, 1974) showed that environmental factors modified the size, arrangement, and distribution of wax crystals on the surface, indicating that the L35bw line was more susceptible to these changes than other lines. In addition, these authors suggested that the observed form of crystals could be the undeveloped form of platelets. In our experiment, drought did not significantly change the diameters of wax tubules in the glaucous rye NILs, and they were in the range of 0.14–0.39 µm (Table 3). This was consistent with the 0.2–0.3 µm range reported by Barthlott et al. (1998) and similar to the results obtained for wheat (0.1–0.3 µm) (Wang et al., 2015b).

Previous studies on flag leaf wax load in glaucous and non‐glaucous wheat varieties (Bi et al., 2016; Guo et al., 2016; Su et al., 2020) clearly indicated a higher wax load of the glaucous line, regardless of treatment and drought stress intensity. In our research, no such clear relationships were found for rye NILs. This indicated that different environmental conditions affected the amount of wax deposited on rye NIL flag leaves (Figures 1 and 3). Higher wax load in the glaucous lines under the drought of 2015 (higher *T*_max_ combined with a lower soil moisture compared with 2016) and in the non‐glaucous line 811bw in the following year (Figures 1 and 3) implied that the non‐glaucous and glaucous rye lines could exhibit different mechanisms of wax deposition in the flag leaf, depending on the prevailing environmental conditions. To date, reports regarding wax protection against excessive water loss suggested that an increased wax accumulation was associated with a lower water loss (Lee and Suh, 2015; Qin et al., 2011; Sajeevan et al., 2017; Seo et al., 2011). Another study reported that glaucousness also contributed to reduced water loss in leaves (Zhang et al., 2015). However, it should be noted that not all studies demonstrated a correlation between reduced water loss and increased wax accumulation, e.g. in alfalfa (Jefferson et al., 2010) and wildrye (Jefferson, 2008). It is also interesting that barley mutants with reduced wax cover were able to grow under limited water conditions (Weidenbach et al., 2015). In this study, no simple correlation was found for rye NILs between the hydration of rye leaves and flag leaf wax content (Table 2). In addition, the higher wax load on the flag leaf of the non‐glaucous NIL 811bw was accompanied by its greater hydration compared with the glaucous NIL 811, and greater differences were noted in 2015 when significantly higher *T*_max_ were recorded during drought (Figures 1 and 3). This indicated different responses of non‐glaucous plants compared with those reported for wheat (Zhang et al., 2015). Patwari et al. (2019) suggested that the relationship of leaf wax cover and water loss could be more complicated than wax accumulation under water deficit, and additionally dependent on the composition of wax cover. In this study, this could be indicated by the fact that the glaucous NIL L35 showed a higher leaf hydration compared with the non‐glaucous NIL under drought in 2016, and only a small difference in wax weight (2%) (Figure 3). This may suggest that in the case of rye plants with different wax phenotype, glaucous or non‐glaucous, wax coating weight on the leaf cannot be unequivocally related to its hydration, and the wax load is under strong influence of environmental conditions. The study of wax composition of the glaucous and non‐glaucous rye NILs also showed strong influence of environmental conditions during the two experiment years both under control and drought conditions (Figures 4, 5, 6, 7). Wax cover composition of rye was recently studied by Sun et al. (2020); however, these authors did not include wax analysis during drought stress. The latter authors identified fractions of alkanes, FAs, alcohols, and esters in flag leaf wax, which were also discussed in the present study; however, unlike Sun et al. (2020), wax of our rye NILs contained a significant proportion of FAs and esters (Figure 3). Despite a similar sampling phase, differences could be due to different growth sites, environmental conditions, or even different cultivars and genotypes (Bi et al., 2015; Lee and Suh, 2015; von Wettstein‐Knowles, 2016). An increase in ester biosynthesis in the leaf wax cover was also observed during drought in Arabidopsis (Patwari et al., 2019). The latter study pointed out that wax esters could contribute to drought resistance, which was also indicated by other reports (Li et al., 2008; Yang et al., 2019). Our research may also be in line with the above findings; however, depending on environmental factors, this correlation could have been disturbed. In this study, a negative correlation was obtained between the FC parameters in 2016 and the content of the ester fraction in epicuticular wax of rye leaves (Table 2). The proportion of ester fraction increased during the drought in all lines, but slight differences were found in pairs, in favour of lines with better FC values, i.e. the non‐glaucous line 811bw and glaucous line L35 (Table 4, Figure 3). Taking into account other fractions, Su *et al*. presented a study on drought‐resistant non‐glaucous wheat line, which exhibited increased alkane biosynthesis under severe drought (Su et al., 2020). Our glaucous rye NILs were characterized by a decrease in hydrocarbon proportion in both years, while Su et al. (2020) reported an increase, but not as high as in the non‐glaucous lines. According to these authors, the higher alkane concentration is one of the mechanisms of non‐glaucous wheat lines to maintain growth and photosynthesis under severe drought. Our research demonstrated that in the case of rye, the non‐glaucous lines increased the proportion of hydrocarbons as a response to drought stress and higher maximum temperatures during drought (2015 vegetation season), whereas a decline was recorded in the glaucous lines (Figure 3). Considering the abundance of individual fractions in individual years, it can be concluded that the differences in environmental conditions could have influenced the involvement of various pathways of wax component biosynthesis. In the first year, the biosynthesis of FAs, directly deposited in the wax layer, and the ester biosynthesis in the acyl reduction pathway of VLCFAs were dominant, while the role of the acyl biosynthesis pathway was predominant in the following year. Interestingly, distinct changes in the percentage composition of the analysed wax fractions were found after drought treatment in 2015. Uniform differences, regardless of the NIL pair, were noted only in the FA fraction, while the percentage of C14:0 and C16:0 short‐chain FAs was higher in the glaucous line, and long‐chain C24:0 in the non‐glaucous line. These differences resulted not only from the glaucous phenotype, but also mainly from a different pair phenotypes (Figures 4, 5, 6, 7). Our results differed from the studies conducted by Su et al. (2020) on non‐glaucous and glaucous Wheat lines, in which biosynthesis of alcohols (acyl reduction pathway) dominated. Wax composition was not the only feature distinguishing the glaucous and non‐glaucous rye NILs, they also had different biochemical, physiological, and morphological characteristics both under optimal and drought conditions (Tables 4, 5, 6). Interestingly, taking into account the results of the wax composition, correlations were found (Table 2) between the analysed physiological and biochemical parameters and the total FAs in the first year (negative correlation) and total esters in the second year (negative and positive correlations). The correlations concerned mainly the content of photosynthetic pigments and tocopherols, whose biosynthesis mainly takes place in chloroplasts, but also the biosynthesis of wax precursors (Colombo, 2010; Kolattukudy, 1970; von Wettstein et al., 1995). The above results and the use of NIL of rye suggested a relationship between genes and wax biosynthesis factors in response to various environmental conditions and drought stress. The literature regarding correlations between physio‐biochemical parameters related to plant response to drought and wax cover composition is limited, but such a relationship was studied by Su et al. (2020).

The results confirmed interaction between the genotype (line), wax layer type (glaucous versus non‐glaucous), and treatment (control versus drought), taking into account significant differences between the studied rye lines in terms of the values of ChlA fluorescence parameters, photosynthetic pigments, and tocopherol contents (Table 1).

Plant exposure to long‐term soil drought stress leads to the generation of toxic reactive oxygen species (ROS), which cause peroxidation of lipid membranes and degradation of nucleic acids and proteins. ROS exert negative effects on a variety of cellular organelles, including chloroplasts (Farooq et al., 2009). Measurements of FC (ChlA fluorescence) parameters and PPCs result in information being obtained about the course of photosynthesis and state of photosynthetic apparatus, in particular PSII, in relation to the damage caused by drought. Apart from the glaucous line 811, higher decreases in FC parameters of other NILs were recorded in 2016, and this could be the result of a several times higher load of flag leaf wax in 2015 (Table 4, Figure 3), which formed a highly photoprotective layer for the photosynthetic apparatus during drought stress. Photoprotective properties of cuticular wax have been described in the literature (Holmes and Keiller, 2002; Sheperd and Griffiths, 2006; Skórska and Szwarc, 2007). In contrast, PSII of the glaucous NIL 811 proved to be very sensitive to drought stress in both 2015 and 2016, despite the significantly higher wax weight during the 2015 drought (Figure 3). The photosynthetic apparatus of NIL 811 was also more susceptible to damage during the 2‐year experiment (Table 4). The protective effect of a heavy wax layer was visible in the other pair of NILs L35 and L35bw during the 2015 drought, while the non‐glaucous line exhibited lower FC parameters in the following year, despite a similar wax load. According to Guo et al. (2016), glaucous wheat NILs protected PSII against drought by accumulating large amounts of wax; however; this study has demonstrated that it is not a simple relationship and environmental factors and wax layer composition likely play a role in this species. Very large amounts of wax could protect PSII both in the glaucous and non‐glaucous rye lines under drought conditions with higher *T*_max_ (2015). However, FC values also depended heavily on individual line pairs, which was apparent in the following year, and they were negatively correlated with the content of the ester fraction (Table 2). The glaucous line L35 had the best PSII performance under drought conditions and the lowest ester content in wax.

Reduction in TChl could be considered a sign of oxidative stress, which causes photo‐oxidation of photosynthetic pigments and TChl degradation (Anjum et al., 2011). Considering chlorophyll function in photosynthesis, such as light collection and power generation reduction, lower TChl under water deficit implies limited photosynthesis potential, and thus primary production metabolism (Anjum et al., 2011). Severe drought stress significantly reduced TChl concentration in rye leaves. Consistently with other studies (Huseynova et al., 2009; Mafakheri et al., 2010; Nikolaeva et al., 2010; Nxele et al., 2017), we have observed a common phenomenon, i.e. a decrease in TChl under water deficit. The substantial degradation (up to 83%) of PPC in the glaucous NIL 811 in both years explained the inability to measure FC in this line and indicated high sensitivity of this NIL to drought‐induced oxidative stress compared with its non‐glaucous counterpart, 811bw (Table 4). In the NIL L35 and L35 pair, differences in PPC content affected PSII functioning when environmental factors were less severe for both lines in 2016. Significantly higher PPC values in the glaucous line could explain its higher FC values (Table 4). Oxidative stress in plant tissue can be alleviated by non‐enzymatic antioxidant systems, including carotenoids (Farooq et al., 2009). They play one of the key roles in the plant antioxidant defence system (Havaux, 1998; Wahid et al., 2007), but they are very sensitive to the damaging effects of oxidative agents. Carotenes present in chloroplasts are associated with the core complexes of PSI and PSII; therefore, protection against damage caused by ROS is so important for the proper functioning of chloroplasts (Farooq et al., 2009). In our experiments, the decrease in Car and individual carotenoid contents (Viol, Lut, and β‐car) under drought (Tables 4 and 5) could be the result of high sensitivity of carotenoids to the harmful effects of oxidative factors. However, it is worth noting that Zea content increased in all NILs during the drought in 2015 with higher *T*_max_, except for the glaucous 811 line, which was characterized by more than 50% decrease in Zea leaf content in both years (Table 5). Zea, through its ROS scavenging ability, can directly reduce oxidative plant damage (Zhao et al., 2014). Tausz et al. (2005) reported an increase in Zea levels in apple leaves during severe drought, while the content of other antioxidants such as β‐car and α‐T decreased. Havaux et al. (2007) demonstrated the ability of Zea in *Arabidopsis* leaves to protect thylakoid membrane lipids at a level comparable with vitamin E, and higher than all other xanthophylls. It was also found in Arabidopsis (Zhao et al., 2014) that Zea accumulation increased plant drought resistance manifested by lower leaf necrosis, decreased lipid peroxidation, and enhanced the photosynthesis rate. In the current study on rye NILs, the results suggested that Zea accumulation could have a great impact on the alleviation of oxidative stress in the non‐glaucous NIL 811bw and both NILs L35 and L35bw under drought, indicating a significantly better PSII function compared with the glaucous NIL 811 showing a strong Zea decrease. Moreover, it should be pointed out that during the drought of 2015, the non‐glaucous line 811bw had three times more Car than the glaucous line 811, which did not produce any seeds in this experiment (Table 4). Considering the better functioning of PSII in the glaucous line L35 during the drought of 2016, significantly higher contents of Car as well as Lut and beta‐carotene should be noted.

Tocopherols and tocotrienols are lipophilic molecules with antioxidant properties, belonging to the group of vitamin E compounds, collectively known as tocochromanols. Their biosynthesis takes place in plastids (Colombo, 2010), although both groups can be present in various tissues and can have different functions (Falk and Munné‐Bosch, 2010; Voll and Abbasi, 2007). Tocopherols may play an important role in plants subjected to drought stress due to their ability to dissipate excessive excitation energy during photo‐oxidative stress (Falk and Munné‐Bosch, 2010; Hernández et al., 2004; Liu et al., 2008). Moreover, tocotrienols show efficient antioxidant activity, protecting membrane lipids from peroxidation (Matringe et al., 2008). The increased content of most of the analysed tocochromanols under drought, both in the glaucous and non‐glaucous lines, could indicate important rye adaptation to water‐deficit conditions and the accompanying photo‐oxidative stress. The non‐glaucous NIL 811bw had more than 10‐fold higher content of tocochromanols in the 2015 drought compared with the glaucous NIL 811, which could also have contributed to lower PSII damage in the NIL 811bw. Considering the high number of reports on an important role of α‐T in photosynthetic tissues during drought (Munné‐Bosch, 2005), it was the most abundant rye tocopherol regardless of treatment in the present study, and its increase under drought was only recorded in the non‐glaucous 811bw and L35bw in 2015; however, it was a slight change compared with other tocochromanols and controls (Table 5). On the other hand, the analysis of data correlation from 2016 showed a positive correlation between the content of β‐T, γ‐T, and δ‐T in leaves and esters in wax, which was not observed for α‐T and was not recorded at all in the previous year (Table 2). The glaucous line 811, accumulated δ‐T and β‐T during water deficits in 2016, and level of these compounds were not increased in the previous year. In addition, despite not being able to measure FC on the last day of drought, the line managed to produce seeds. Moreover, during the drought of 2016, the non‐glaucous NIL L35bw accumulated more δ‐T, β‐T, and γ‐T, but the glaucous line L35 was characterized by higher FC values and yield (Table 5). This indicated a different contribution of each tocochromanol to PSII protection in individual rye lines, implying that other compounds such as carotenoids could play a more important role in this process. Greater changes in tocopherol levels due to drought in 2016 could be associated with increased MDA levels in most lines, with the exception of the non‐glaucous L35 line. MDA is one of the final products of lipid peroxidation (Cunhua et al., 2010; Gharibi et al., 2016; Wang et al., 2012) and is accumulated largely in plants subjected to drought stress (Cunhua et al., 2010; Gharibi et al., 2016; Mirzaee et al., 2013). Lower FC values during the drought of 2016 displayed by the non‐glaucous line could be more likely due to the lower amounts of Car, while higher levels of tocopherols protected it from increased lipid peroxidation compared with the glaucous line L35. The glaucous NIL 811 was again the most sensitive line, but the MDA level in the 2016 drought did not differ significantly between the NILs 811 and 811bw, which could also suggest a role for tocopherol accumulation in preventing further damage in this glaucous line.

It has been proven that the accumulation of phenolic compounds may be a plant adaptation to drought stress (Basu et al., 2010; Dixon and Paiva, 1995; Hura et al., 2007, 2013; Rosales et al., 2012; Treutter, 2005) and it increases protection of the photosynthetic apparatus during dehydration (Hura et al., 2007). In our study, soil drought stress caused different changes in TPC levels in the glaucous and non‐glaucous lines that were strongly dependent on environmental factors. Under the drought of 2015, when *T*_max_ was higher, only the glaucous NILs had a reduced TPC content, but the non‐glaucous NIL 811bw was the only one that had a higher TPC content than its glaucous counterpart. In the following year, both NILs, 811 and 811bw, did not differ significantly and both had lower TPC, and it was consistent with a decrease reported by Czyczyło‐Mysza and Myśków (2017). Interestingly, other NILs showed contrasting trends, and only the glaucous NIL L35 exhibited a significant increase in TPC level, while its non‐glaucous equivalent NIL L35bw showed a significantly higher TPC level than NIL L35 despite a decrease in TPC (Table 5). According to Hura et al. (2009), the TPC increase under water deficit in the glaucous NIL L35 distinguished this line from other NILs, as there were reports that showed increased TPC levels could be an additional criterion for selecting drought‐resistant genotypes. It was also important due to the highest PSII efficiency and grain yield among other NILs, including the counterpart of NIL L35, the non‐glaucous line L35bw.

During abiotic stresses in plants, soluble sugars participate in the osmotic adjustment, free radical scavenging, and protein stabilization (Ingram and Bartels, 1996; Jouve et al., 2004; Silva and Arrabaça, 2004). Thus, many studies on drought stress in plants demonstrated an increase in the synthesis of leaf‐soluble sugars (Homayouni and Khazarian, 2014; Hura et al., 2016; Marcińska et al., 2013). However, a significant decrease (25–77%) in SSC was demonstrated in the leaves of the tested glaucous and non‐glaucous rye NILs under soil drought stress during both years (Table 5). Our results were consistent with the report of Czyczyło‐Mysza and Myśków (2017), and the observed decrease in SSC could result from a very severe water deficit (Pinheiro and Chaves, 2011). Similar to the analysis of previous parameters, the glaucous 811 line proved to be more sensitive than the non‐glaucous 811bw line, as the highest decline in SSC was recorded for this line regardless of the year. Significantly lower SSC values were recorded for the glaucous L35 line during the 2015 drought; however, opposite results were obtained in the second year, the non‐glaucous NIL L35 exhibited a two times higher SSC decrease than the glaucous line L35 (Table 5). Once again, the effect of environmental conditions and highly differentiated reactions of the examined NIL pairs could be observed. Our results and the findings of Czyczyło‐Mysza and Myśków (2017) could indicate a different rye response to drought in terms of the accumulation of leaf‐soluble sugars.

Long drought also significantly affected the agronomic characteristics of rye plants, resulting in a lower value of yield components, biomass of the above‐ground plant parts and its height (Table 6). During both experiments, the 811 and 811bw pair experienced the highest decreases (even up to 100%) of those traits, indicating that this pair was less resistant to water deficit than the pair of the lines L35 and L35bw. The decrease in yield components due to water deficit has been widely described in the literature (Cyganek, 2018; Czyczyło‐Mysza and Myśków, 2017; González and Ayerbe, 2010; Guo et al., 2016; Myśków et al., 2018; Talebi, 2011). All measured components exhibited some differences between the glaucous and non‐glaucous lines. The glaucous line L35 produced more grains (32%) and had a higher TGW (19%) than the non‐glaucous L35bw under drought during the second experiment (Table 6); this was also demonstrated previously in glaucous crops, such as barley and wheat, where glaucous plants had a higher grain yield (Baenziger et al., 1983; Guo et al., 2016; Johnson et al., 1983; Merah et al., 2000; Richards et al., 1986). Moreover, the glaucous line L35 had the highest GW and TGW among the tested lines under drought (during both experiments). Febrero et al. (1998) noted that the yield of barley glaucous plants was increased in a water‐limited environment. The relation of glaucousness with grain yield was also confirmed by quantitative trait loci analysis in wheat carried out by Hill et al. (2013), who found two regions on chromosome 7A that simultaneously affected glaucousness and grain yield, indicating a correlation between these traits. Merah et al. (2000) concluded that the drought introduced earlier in wheat caused significant differences in grain yield in favour of glaucous plants, while higher biomass production was recorded in the non‐glaucous phenotype when the drought was introduced later and the plants had time for stress‐free biomass production. In our research, the biomass of the above‐ground parts of rye plants in the glaucous and non‐glaucous lines did not significantly differ in most cases or was significantly lower in the non‐glaucous line. The physiological basis for differences in ^13^C isotope discrimination (δ^13^C) in C_3_ plants is related to the variation in the internal CO_2_ to ambient CO_2_ concentration ratio. According to Farquhar et al. (1989), heavier and less abundant ^13^C isotope discrimination occurred in C_3_ plants during photosynthetic gas exchange when CO_2_ was incorporated into plant biomass, which, among others, was associated with a greater preference of more abundant and lighter ^12^C by Rubisco involved in carbon fixation during photosynthesis. A reduced discrimination by the Rubisco enzyme is observed during closed stomata, when the amount of CO_2_ in chloroplasts is lower. Higher δ^13^C values correspond to a higher water use efficiency. The δ^13^C has been proposed as a selection criterion for cereal grain yield under dry conditions, because δ^13^C is directly related to transpiration efficiency and integrates it throughout the growth period of the sample tissue. Positive correlations were found between the δ^13^C values of leaf and grain yield (Kottmann et al., 2014; Merah et al., 2000; Monneveux et al., 2004); however, Monneveux et al. (2006) reported the correlation only for grain δ^13^C. So far, higher δ^13^C values have been reported in glaucous plants growing in different water regimes (Febrero et al., 1998; Merah et al., 2000; Monneveux et al., 2004); however, there are also studies in which such relationships were not found (Adamski et al., 2013; Frizell‐Armitage, 2016). This suggests that glaucousness may not simply be correlated with higher δ^13^C values. This was also demonstrated in our study. The glaucous NIL 811 had significantly higher leaf δ^13^C than the non‐glaucous NIL 811bw, but the glaucous NIL was also very susceptible to drought and did not produce any seeds in 2015, had significantly lower TGW than 811bw in the following year, and less hydrated leaves than the non‐glaucous counterpart (Table 6). In contrast, the other pair of NILs showed no differences in the δ^13^C value and did not differ in yield during the 2015 drought, while the higher δ^13^C value in the glaucous line L35 during the 2016 drought corresponded to a higher number and weight of seeds and flag leaf hydration in comparison with the non‐glaucous NIL L35bw. Our results did not show a simple relationship between high δ^13^C values and glaucousness, but this could be an interesting subject for further analysis in L35 and L35bw NILs.

## Conclusions

Environmental factors had a great effect on the response of the studied lines to drought in individual years, both in terms of physiological and biochemical reactions and the composition of epicuticular leaf wax. The use of unique glaucous and non‐glaucous rye NILs as plant material, which by definition differ only in wax coating, allowed us to characterize them and attempt to investigate the impact of the type of wax cover and soil drought stress on the analysed physiological and biochemical parameters and agronomic characteristics such as yield. Given the higher light reflectance values for glaucous plants reported in the literature, leaf glaucousness is useful in preventing photo‐damage due to high light intensity that occurs simultaneously with drought. This may imply that glaucous lines should be less susceptible to photosynthetic apparatus damage caused by these conditions. However, our results suggest that these correlations may not be as straightforward when comparing yield and physiological and biochemical status of the tested NIL pairs. In addition, different pairs displayed significantly different responses to drought, making it impossible to state unequivocally that only the glaucous or non‐glaucous plants are better adapted to water shortage. Further studies on L35 and L35bw NILs will provide more consistent answers on the effect of glaucousness on plant responses to drought. Our study showed that wax accumulation during drought was not correlated with higher leaf hydration and glaucousness. Drought stress in combination with higher *T*_max_ (2015), had a stronger effect on wax cover composition; however, these changes differed not only between the non‐glaucous and glaucous lines, but also between the NIL pairs, which indicated high variability in the characteristics of wax cover composition in rye. An important discovery of this work is the demonstration of the correlation between the components of rye leaf wax and physiological and biochemical parameters of rye NILs. This correlation was both positive and negative, while its direction differed depending on the analysed year. Interestingly, the study showed a correlation between wax components and the content of photosynthetic pigments and tocopherols. The above results as well as the use of NIL of rye, suggested a relationship between wax biosynthesis genes and plant response to various environmental conditions and drought stress. During the drought in 2016, a higher proportion of hydrocarbons in wax cover was found in the non‐glaucous 811bw and glaucous L35 lines, which differed from their counterparts in higher yields; however, the correlation coefficients did not prove that. It is possible that further research using the latest molecular biology techniques is needed to extend this study.

An important success of the present work is the extensive physiological and biochemical analysis of the glaucous and non‐glaucous lines using NIL pairs that demonstrated different responses to drought stress. In this study, the results obtained for the analysed glaucous and non‐glaucous lines could suggest that carotenoids, in particular Zea (during more difficult environmental conditions) and tocochromanols were of great importance among the tested antioxidant molecules. It could be assumed that during the 2015 drought with higher *T*_max_, a significantly higher content of these molecules, particularly carotenoids, in the non‐glaucous NIL 811bw than in the glaucous NIL, protected it from massive PSII damage, which occurred in the glaucous line 811 that did not produce any seeds at maturity. During the drought period in the following year, the glaucous NIL 811, despite immeasurable FC values, produced seeds, and the lower level of accumulated MDA compared with the previous year could have been caused by the accumulation of other tocochromanols than in 2015. The NIL pair L35/L35bw did not show differences in yields during the 2015 drought, while the following year was characterized by higher GN and GW in the glaucous NIL L35. The differences were not as significant compared with the NIL pair of 811 and 811bw and, apart from the supposed role of carotenoids, this pair requires further research concerning drought response. An interesting issue was the significantly higher value of δ^13^C in the glaucous L35 line, while the glaucous NIL 811 did not show such a tendency.

There is premise for further biochemical, physiological, and genetic studies that will complement our results. Our research shows how complex the phenomenon of wax cover occurrence in plants is, and that there is still great potential in this area to draw strong conclusions about the role of the cuticle in drought resistance.

## EXPERIMENTAL PROCEDURES

### Plant material

**Figure 8:**
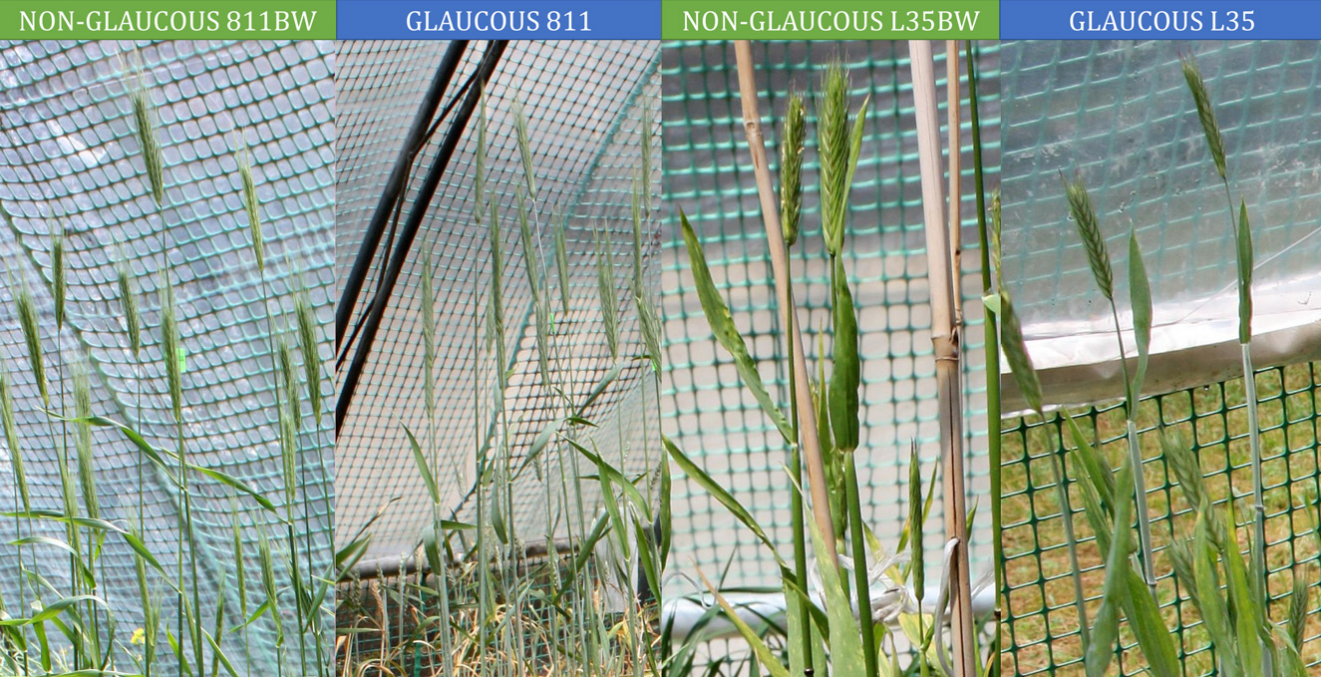
Near‐isogenic lines of rye: glaucous 811 and L35 and non‐glaucous 811bw and L35bw.

The plant materials included two pairs of rye NILs derived from two different mapping populations of recombinant inbred lines (RIL) at the Department of Plant Genetics, Breeding and Biotechnology, West‐Pomeranian University of Technology in Szczecin (Table 7, Figure 8). Each pair of inbred lines (811; 811bw and L35; L35bw) consisted of a typical line and one with a recessive mutation disrupting the formation of the proper wax coating. Mutant plants appeared to be devoid of wax, and leaves, stalks and ears had an intense green colour, rather than the typical bluish coating, which was later dubbed ‘non‐glaucous’ and marked with the suffix ‘bw’ (Table 7, Figure 8). A typical line was described as ‘glaucous’.

**Table 7:** Genetic and phenotypic characteristics of rye near‐isogenic line pairs

Pair of lines	Mutation	Pedigree	Characteristic
811^a^ 811bw^b^	waxM	Interline hybrid S120×S76. A non‐glaucous plant appeared in RIL population S120×S76 at F7	Standard height, narrow leaves, limp plants
L35^a^ L35bw^b^	waxR	Interline hybrid DS2×RXL10. A non‐glaucous plant appeared in RIL population DS2×RXL10 at F8	Dwarf, wide leaves, stiff plant

^a^ Glaucous.

^b^ Non‐glaucous.

A pair of sublines (NILs), labelled as L35 and L35bw, was derived from the interline hybrid Ds2×RXL10, when generating a population of RILs, RIL‐L. A non‐glaucous recessive mutation appeared in the RIL‐L at the S8 level, as single plants with the genotype L35. Offspring of non‐glaucous plants was treated as the line L35bw. Its glaucous counterpart (non‐segregating, homozygous subline) was named L35. The genetic similarity of the L35 and L35bw lines was analysed using the RAPD‐PCR method with the use of 678 primers, and was 0.95.

NIL 811 was derived from the interline hybrid S120×S76. In the population of RIL S120×S76, named RIL‐M, a non‐glaucous plant appeared at the S6 level. This plant with a recessive mutation (M12bw) was crossed with a plant with a normal waxy cover, phenotypically similar to M12bw (M14). Segregating RILs were selected from the M14×M12bw hybrid after five generations of inbreeding. The chosen pair of sublines (non‐glaucous 811bw and non‐segregating glaucous 811) were treated as a pair of NILs. The genetic similarity of NIL 811 was not analysed.

### Drought stress experiment

The pot experiment was conducted twice in 2015 and 2016 to study the effect of drought stress on physiological and biochemical parameters of rye NILs at the *The Franciszek Górski* Institute of Plant Physiology of Polish Academy of Sciences in Kraków. Rye grains coated with fungicide powder were germinating for 2 days in plastic containers filled with sterilized perlite in the dark at 25–26°C. Germinating seeds of four NILs were vernalized in containers with perlite at 3–4°C with an 8‐h photoperiod for 74 days. The seedlings were transferred to pots (Ø15 cm, 20 cm height) filled with sieved soil (composed of equal volumetric proportions of gardening soil with peat substrate and sand) and placed in an open vegetation tunnel, where seedlings were grown in individual pots with at least 16–20 replicates (one seedling/pot). The soil was watered with the same volume the day before planting the seedlings. Initially, all plants were grown in conditions of optimum soil moisture content, and after transitioning into the shooting phase, they were divided into two groups of different soil moisture, each group within the line consisted of at least eight to 10 replicates. Each pot contained a single plant as a replicate. The first group was the control, in which plants grew under optimum soil moisture conditions (approximately 70% field water capacity), while the second group was exposed to drought stress (approximately 30% field water capacity) for a period of 3 weeks. During the 3‐week drought period, control plants were watered each day with the same volume per day per pot (50 or 100 or 200 ml), which was adequate for the overall viability and soil appearance of plants on a given day. Plants subjected to drought stress were not watered throughout the drought period. The plants grew in natural daylight and air temperature, characteristic of the spring–summer period (May–August). During the experiments, soil water content was inspected using a moisture meter [volumetric water content measurement (%); Campbell Scientific 620, Inc., Shepshed, Leicestershire, UK]. The volumetric water content measurements were conducted once a week during the drought of 2015 and 12 times during the drought of 2016 (presented as an average for each of 3 weeks). Photochemical activity of PSII (fluorimeter Handy PEA; Hansatech, Kings Lynn, Norfolk, UK) was measured on the last day of drought. Subsequently, flag leaves of the main shoot were cut off, frozen, and stored for biochemical analysis. Afterwards, the plants grew under a plastic shelter until full maturity. Meteorological conditions in Cracow during the spring–summer period were monitored and collected by a Vaisala WXT520 automatic meteorological station operated by the Environmental Physics Group, Faculty of Physics and Applied Computer Science (AGH University of Science and Technology, Cracow) (Figure 1).

### Flag leaf wax cover analysis by SEM

The analysis of the flag leaf of rye NILs using an SEM was outsourced to an external institution (Laboratory of Scanning Microscopy of Biological and Geological Sciences at the Institute of Geological Sciences of the Jagiellonian University equipped with a Hitachi S‐4700 microscope and the NORAN Vantage microanalysis system). Leaf samples were collected only in 1 year of the experiment. Plant tissue samples approximately 5 mm × 5 mm in size were taken on the last day of drought from both control and drought‐treated samples. The samples were air‐dried and subsequently placed in a desiccator, as the SEM procedure required water‐free samples. At the external institution, the samples were sprayed with gold and analysed under an SEM microscope where microphotographs of adaxial and abaxial leaf blades were taken at 10 000× magnification.

### Leaf hydration and quantitative analysis of wax fractions

The wax fraction was collected from the fully developed flag leaves. Leaves were gently immersed in 20 ml of dichloromethane (DCM) in a long, screw‐top glass vial. After washing, rye leaves were transferred to a separate set of plastic tubes, weighed, lyophilized for 72 h and re‐weighed to obtain their dry mass (semi‐microanalytical scale MYA 31.4Y; RADWAG, Radom, Poland). Leaf hydration was calculated based on the fresh and dry mass percentage. DCM fraction was evaporated under N_2_ at 40°C (TurboVap; Zymark, Hopkinton, MA, USA) and the residual wax fraction was weighed (0.01 g accuracy). The analyses of wax fraction were based on methods described by Dove and Mayes (2006) and Wirth and Sessions (2016). The wax fraction was saponified overnight with 1 m NaOH in 88% methanol in a shaking water bath at 70°C. The samples were then neutralized with concentrated HCl and triple extracted with n‐hexane. Pooled n‐hexane layers were concentrated to 1 ml and separated into four fractions of increasing polarity on SPE cartridges (BondElut NH2, Agilent, Santa Clara, CA USA); before that, a 30‐μl aliquot was collected for phytosterol analysis. Eluted fractions consisted of hydrocarbons (1 ml hexane), esters and ketones (1 ml 4:1 hexane/DCM), alcohols (1 ml 9:1 DCM/acetone), and carboxylic acids (1 ml 3% formic acid in DCM). In 2015 a pooled biological wax sample per line under each treatment was collected, whereas in the following year there were three pooled biological wax samples per line under each treatment.

#### Profiling of hydrocarbons

Samples were evaporated and reconstituted in 100 μl of carbon disulphide. Hydrocarbons analyses were carried out using a 7890A GC system (Agilent) with a flame‐ionization detector. Separation was conducted on DB‐5HT (30 m × 0.32 mm × 0.1 μm; Agilent), H_2_ was the carrier gas at a flow rate of 3 ml min^−1^, split ratio of 8:1, the injection port was set to 350°C, and column temperature was as follows: 50°C increased to 250°C at a rate of 30°C min^−1^, then from 250 to 350°C at a rate of 75°C min^–1^ and held for 2 min, after which the column was flushed at 8 ml min^−1^ for 3 min. The injection volume was 1 μl. Identification of the hydrocarbons was based on injection of commercial standards (Supelco, Bellefonte, PA, USA). Tetradecane was used as an internal standard (ISTD).

#### Profiling of the ester fraction

The ester fraction after evaporation was transesterified with 0.5 ml 10% H_2_SO_4_ in methanol for 2 h at 60°C, then, after neutralization, methyl ester fraction was extracted to n‐hexane with saturated Na_2_CO_3_. Heptadecanoic acid was used as ISDT. Analyses were carried out on a 7890A GC‐FID system. The parameters were as follows: HP‐88 (60 m × 0.25 mm × 0.2 μm; Agilent) column, carrier gas H_2_ at 2 ml min^−1^, split ratio of 10:1, the injection port was set to 250°C and column temperature was as follows: 120°C increased to 175°C at a rate of 10°C min^−1^, hold for 8 min, then from 175 to 210°C at a rate of 5°C min^−1^ and held for 3 min, after which, the column was flushed at 8 ml min^−1^ for 3 min. The injection volume was 1 μl. Identification of methyl esters was based on injection of the commercial FAME standard (Supelco).

#### Primary alcohol fraction profiling

The primary alcohol fraction was evaporated and reconstituted in 100 μl of carbon disulphide. Sufficient separation was achieved without derivatization under the conditions described for hydrocarbons. Identification was based on the analyses of the commercial fatty‐alcohol standard kit (Chromadex, Irvine, CA, USA). Tetradecane was used as an internal standard (ISTD).

#### Carboxylic acid profiling

The carboxylic acid fraction was evaporated, and then esterified and analysed under the exact conditions described for the ester fraction. Heptadecanoic and nervonic acid were used as an ISTD. Identification of methyl esters was based on injection of the commercial FAME standard (Supelco).

### Photochemical activity of PSII

The kinetics of ChlA fluorescence was determined with a Handy PEA fluorimeter (Hansatech, King’s Lynn, UK). All measurements were performed in the middle of the flag leaf after shading the clip for about 20 min. PEA analysers allow to conduct the OJIP test (Strasser et al., 2000). On this basis, the following parameters ChlA fluorescence kinetics were calculated and analysed: *F*_v_/*F*_m_ (maximum quantum yield of primary PSII photochemistry), PI (overall performance index of PSII photochemistry), ABS/CSm, ETo/CSm, DIo/CSm, TRo/CSm, RC/CSm.

### Biochemical measurements

After 72 h of lyophilization all collected leaves were pulverized in a mixing mill (MM 400; Retsch, Kroll, Germany) at a maximum frequency (30 Hz). A micro‐analytical balance was used for weighing the samples. The analysis required precisely weighed homogenate samples (5 mg), which were extracted in 1.5 ml of 96% ethanol for 15 min in a mixing mill at a frequency of 30 Hz and then centrifuged at 21 000 *g* for 5 min. Subsequently, the extracts were used to conduct all analysis, i.e. PPC, spectrophotometric analysis of SSC and TPC.

### PPC

TChl and Car were estimated according to the modified method by (Lichtenthaler and Wellburn, 1983). An aliquot of ethanolic extract (100 μl) was added to a 96‐well microplate, and the absorbance was measured at 470, 648, and 664 nm using a Synergy II Microplate Reader (BioTek Instruments, Winooski, VT, USA). The concentrations of TChl and Car were calculated according to the extinction coefficient given in the equations of Lichtenthaler and Buschmann (2001). The reported concentration value is an average of five biological replications, each consisting of two technical replications and expressed as the TChl/Car content per milligram of leaf dry weight (µg mg^−1^ DW).

### Analysis of tocopherols, tocotrienols, and carotenoids

Tocopherols (α, γ, β, δ), tocotrienols (α, β, δ) and carotenoids (Viol, Lut, Zea, β‐car) were measured based on a modified method described by Surówka et al. (2016). The samples were extracted in 1 ml ethanol/acetone/methanol/2‐propanol (8/3/3/1 v/v) containing 1% butylated hydroxytoluene solution by shaking in a water bath at 75°C for 15 min. Then, 250 µl of 80% KOH was added and the extraction was continued for 30 min. Next, the samples were diluted with 3 ml H_2_O and purified by double extraction with n‐hexane. Combined n‐hexane layers after in vacuo evaporation (RotaVapor, Buchi, Switzerland) were reconstituted in 0.1% butylated hydroxytoluene solution in methanol/DCM (3/1 v/v) before HPLC separation. An Agilent 1260 UHPLC binary system with diode array detectors and fluorescence detectors were used. Separation was carried out on an Ascentis Express RP‐Amide (3 × 150 mm; 2.7 µm; Supelco) column at 0.8 ml min^−1^, 60°C and a linear gradient of (A) 0.5% formic acid in acetonitrile/H_2_O (6/4 v/v) and (B) 0.5% formic acid in 2‐propanol/acetonitrile (9/1 v/v), from 40% to 100% of (B) for 15 min. Tocochromanols were detected by fluorescence detector at an excitation wavelength of 295 nm and an emission wavelength of 330 nm, while carotenoids were detected by diode array detector at 450 nm. The identity and quantity of monitored compounds were confirmed by comparison with data obtained for pure standards under identical conditions as for the samples. The standards of α‐, γ‐, δ‐T and α‐, γ‐, δ‐tocotrienol, solvents, and reagents were purchased from Sigma‐Aldrich (Poznań, Poland). Carotenoid standards were acquired from DHI Lab Products (Horsholm, Denmark). The analyses were performed in three replicates per line under each treatment and the results were expressed as tocochromanol/carotenoid content per milligram of leaf DW (ng mg^−1^ DW).

### SSC

Spectrophotometric analysis of SSC was conducted according to Dubois et al. (1951). Ethanolic extract (50 μl) was diluted with 150 μl of water and then 200 μl of 5% water phenol solution and 1 ml of concentrated sulphuric acid were added; the whole reaction mixture was vortexed immediately after adding the reagents. The samples were subsequently transferred to 96‐well plates and absorbance at 490 nm was measured. Sugar content was estimated using the standard curve prepared for glucose. SSC analysis was performed in five replications per line under each treatment and the results were expressed as SSC per milligram of leaf DW (µg mg^−1^ DW).

### TPC

TPC was measured according to the modified method of Singleton et al. (1999). An aliquot of ethanolic extract (100 μl) was diluted in 0.5 ml of deionized water and 0.2 ml of Folin‐Ciocalteu reagent, and after 10 min, 0.7 ml saturated Na_2_CO_3_ was added. The samples were mixed after 2‐h incubation in the dark, centrifuged, and transferred to 96‐well plates. The absorbance at 765 nm was read using a microplate reader (Synergy II; BioTek). Gallic acid was used as a standard. The analysis of TPC was carried out in five replications per line under each treatment and the results were shown as TPC per milligram of leaf DW (µg mg^−1^ DW).

### Lipid peroxidation product: MDA content

MDA content was determined according to Heath and Packer (1968). Plant tissue was homogenized using a ball mill (Retsch MM400). The accurately weighed samples were subsequently extracted with 10% trichloroacetic acid; the homogenate was centrifuged and the supernatant was mixed with 0.5% thiobarbituric acid in 10% trichloroacetic acid. After 30 min of incubation at 95°C, the samples were transferred to 96‐well plates. Absorbance was read at 532 nm using a microplate reader (Synergy II; BioTek). Pure MDA was used as a standard. Oxidative damage during drought was estimated based on MDA content per milligram of leaf DW (µg mg^−1^ DW).

### ^13^C carbon isotope discrimination (δ^13^C) analysis

^13^C carbon isotope discrimination (δ^13^C) was carried out in flag leaves after the end of the drought period. This analysis was outsourced to an external institution (Isotope Dating & Environment Research Laboratory, Institute of Geological Sciences, Research Centre, Warsaw). This analysis measures the sum of all drought effects over the entire growing period of a plant. The discrimination of ^13^C carbon isotope is expressed in ‰ relative to V‐PDB standard.

### Determination of yield components, above‐ground plant biomass and plant height

Plants reaching the stage of final maturity were analysed. The height of a single plant was obtained by measuring the length of the main stem and spike with awns, and then the whole plant was cut just above the soil surface and dried. The material was weighed and the following measurements of the above‐ground plant parts and the crop were performed: yield indicators (GN, GW, and TGW) and the dry weight of the above‐ground parts of a single plant.

### Statistical analysis

The mean ± standard error, anova data analysis, and Pearson’s correlation analysis were obtained and carried out using the statistica software (v.13.1). *Post hoc* multiple comparison was carried out using Duncan’s multiple range tests to compare mean values of agronomic, physiological, and biochemical traits.

## Author contributions

IMCM and BM initiated, conceived, and coordinated the experiments; MD and KL performed chromatographic and biochemical analyses; IMCM, KL, and AN took physiological measurements; JB collected, analysed, and described the meteorological data; KL analysed data and wrote the paper; IMCM, BM, MD, and MG edited and supervised the paper. All authors have read and agreed to the published version of the manuscript.

## Conflict of interest

The authors declare that they have no competing interests.

## Supporting information


**Table S1**. Daily average (*T*_mean_), minimum (*T*_min_), and maximum (*T*_max_) temperature, and daily average (RH_mean_), minimum (RH_min_), and maximum (RH_max_) relative humidity during the spring–summer period in the 2‐year experiment. Orange highlight – drought periods.

**Table S2**. Correlation coefficients between leaf epicuticular wax (wax load, fraction contents and their individual components) and biochemical and physiological parameters, yield, above‐ground plant biomass, and plant height of rye near‐isogenic lines obtained.

## Data Availability

Data supporting the findings of this work are available in the manuscript or in the Supporting Information files. All relevant data can be found within the manuscript and its supporting materials.
